# Long-term recovery behavior of brain tissue in hydrocephalus patients after shunting

**DOI:** 10.1038/s42003-022-04128-8

**Published:** 2022-11-08

**Authors:** Seifollah Gholampour, David Frim, Bakhtiar Yamini

**Affiliations:** Department of Neurological Surgery, University of Chicago, Chicago, IL USA

**Keywords:** Computational biophysics, Neuroscience, Hydrocephalus

## Abstract

The unpredictable complexities in hydrocephalus shunt outcomes may be related to the recovery behavior of brain tissue after shunting. The simulated cerebrospinal fluid (CSF) velocity and intracranial pressure (ICP) over 15 months after shunting were validated by experimental data. The mean strain and creep of the brain had notable changes after shunting and their trends were monotonic. The highest stiffness of the hydrocephalic brain was in the first consolidation phase (between pre-shunting to 1 month after shunting). The viscous component overcame and damped the input load in the third consolidation phase (after the fifteenth month) and changes in brain volume were stopped. The long-intracranial elastance (long-IE) changed oscillatory after shunting and there was not a linear relationship between long-IE and ICP. We showed the long-term effect of the viscous component on brain recovery behavior of hydrocephalic brain. The results shed light on the brain recovery mechanism after shunting and the mechanisms for shunt failure.

## Abstract (web summary)

Data from hydrocephalus patients, along with simulated data, sheds light on the mechanisms of long-term brain recovery after shunt surgery.

## Introduction

Hydrocephalus is caused by the accumulation of excess cerebrospinal fluid (CSF) due to an imbalance between CSF production and absorption^1^. The prevalence of hydrocephalus in adults is 11/100,000^2^, and its incidence is increasing^3^. Shunt surgery is the primary and mainstay of hydrocephalus treatment^4–7^. However, shunt efficiency is variable^4^, and despite recent advances in technology, the shunt failure rate in adults remains high (up to 32%)^8^. Neurosurgeons cannot still predict shunt outcomes, even in patients with similar clinical conditions. Medical history, clinical symptoms, and non-invasive imaging are the main tools for evaluation of hydrocephalus. However, history and clinical symptoms are not solid evaluation indexes^9,10^. For instance, because of clinical overlap, some types of hydrocephalus are misdiagnosed as Alzheimer’s or Parkinson’s disease^11–13^. Neuroimaging may also be inexact, for example, the sensitivity of computed tomography (CT) characteristics to shunt response is only 46% in some types of hydrocephalus^14^. On the other hand, intracranial pressure (ICP)^15^, trans-mantle pressure, CSF volume^16^, and CSF velocity and flow rate^17^, as well as changes in CSF circulation and impairment mechanism^18,19^, are not adequate comprehensive evaluation indexes. Important reasons for the uncertainty in shunt outcome may be the complex behavior of the brain when exposed to elevated ICP as a result of hydrocephalus and the unpredictable recovery behavior of brain tissue after shunting.

Numerous experimental studies have expanded our understanding of brain tissue behavior by experimenting on the brains of healthy rats^20–23^, pigs^24–30^, dogs^31,32^, and sheep^33,34^. Despite acquisition of valuable information from these animal studies, a drawback can be the translation of animal findings to human physiology^35,36^. In addition, the critical issue with the majority of experimental human/animal studies is that they have been performed on healthy subjects^37–40^, not hydrocephalus patients. Very few studies have examined the hydrocephalic brain such as an in vivo study by Shulyakov et al who worked on rat hydrocephalus^41^.

Recently we showed that an important aspect of experimental assessment of hydrocephalic brain is the issue of the length of time that brain tissue is under elevated ICP^42^. This is a parameter that has been poorly studied. Firstly, hydrocephalus is often not acute with many patients presenting having had raised ICP for extended periods of time^17,42^. To accurately model this, it would be important to have a model where increased pressure on the brain is maintained for a long time^42^. Second, previous studies proved the long-term (even more than one year) changes in CSF dynamics and morphometric parameters for adult non-communicating hydrocephalus and pediatric hydrocephalus patients after shunting^17,43,44^. In addition, shunt failure is often seen many months or years after shunting^7^. Therefore, evaluation of brain recovery behavior over an extended period of time after shunting is of great importance.

To study the above concerns, we studied the MRI images of healthy subjects and communicating hydrocephalus patients in pre-shunting and 7 stages after shunting. Then we calculated the changes in volume, strain, and creep of CSF and brain, as well as ICP, exerted force on the brain tissue, brain stiffness, and intracranial elastance (IE) non-invasively using fluid-structure interaction (FSI) simulation to evaluate the brain recovery behavior of hydrocephalus patients over 15 months after shunting.

## Results

### Data validation

**Fig. 1:**
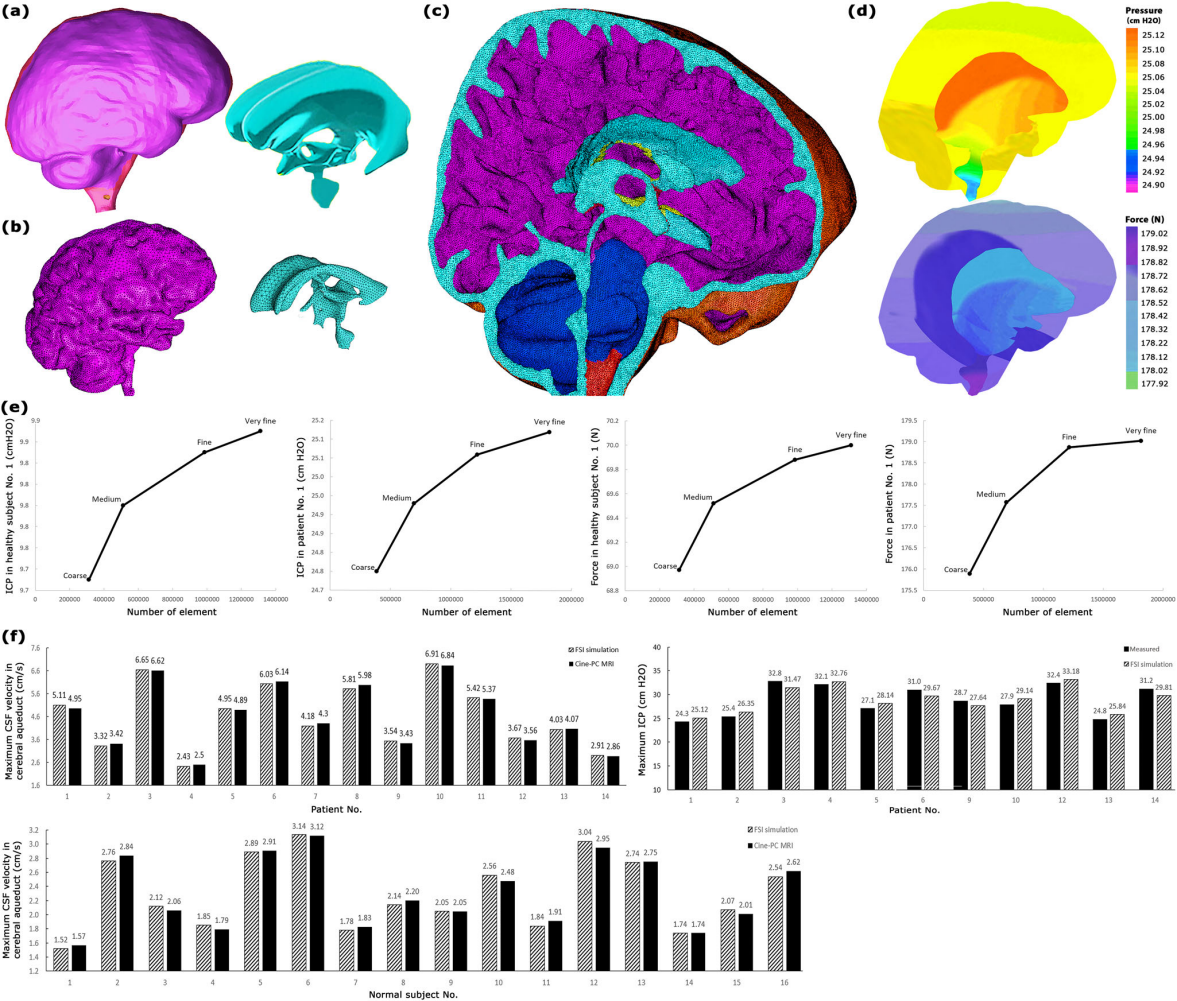
Computer simulation and data validation. **a** 3-D models of the outer layer of subarachnoid space and ventricular system (not to scale) for patient No. 1 before shunting. **b** Meshed models of brain and ventricular system (not to scale) for patient No. 1 fifteen months after shunting. **c** meshed model of the head substructure for patient No. 1 fifteen months after shunting. Turquoise and purple areas represent the fluid domain (CSF space: ventricular system and subarachnoid) and solid domain (brain), respectively. The borders and interfaces between turquoise and purple areas are the FSI boundaries. **d** The FSI simulation results for ICP and force for patient No. 1 before shunting. **e** Grid independence study. **f** Validation of the maximum CSF velocity in the cerebral aqueduct and maximum ICP which were measured using FSI simulation. Raw data for panel f is included in Supplementary Table 1. CSF cerebrospinal fluid, ICP intracranial pressure, FSI fluid-structure interaction, Cine-PC MRI cine phase-contrast magnetic resonance imaging.

After applying the computer simulation process including 3-D modeling (Fig. 1a), mesh generation (Fig. 1b, c), FSI simulation (Fig. 1d), and grid independence study (Fig. 1e), the CSF dynamic parameters were calculated non-invasively. The accuracy of the CSF velocity and ICP calculated using this method was verified in our prior study^45^. A method to validate the correctness of computer simulations is to compare one of the simulation results with in vivo data. Based on this approach, for data validation, previous computer simulations compared CSF velocity measured by Cine phase-contrast magnetic resonance imaging (cine-PC MRI) with the corresponding velocity calculated by FSI simulation^46–50^. In the present study, we also compared the CSF velocities in the cerebral aqueduct of all patients and healthy subjects which were measured by cine-PC MRI (second MRI output), with similar data calculated by FSI simulation for data validation (Fig. 1f, Supplementary Table 1 and Table 1). The maximum differences between CSF velocities in the cerebral aqueduct in all healthy subjects and patients were less than 3.3%. PC MRI does not permit imaging at cardiac frequency, whereas CSF and brain pulsations exhibit cardiac frequency behavior^51,52^, and consequently, measurement of CSF velocity by cine-PC MRI maybe accompanied by some errors^53^. Hence, the aforementioned differences between our results and Cine-PC MR velocity results do not mean that our results had necessarily a 3.3% error. In addition, we compared calculated ICP with experimental ICP. A hole with a 2 mm diameter was created in the skull. An ICP micro sensor (Codman MicroSensor, Johnson and Johnson, Raynham, Massachusetts, USA) was inserted at 1.5–2 cm depth from the outer layer of the skull. Then the ICP was measured for 11 patients before the shunting stage. The difference between calculated and experimental ICP was less than 4.5% (Fig. 1e). We also compared our findings for healthy subjects and hydrocephalus patients in the pre-shunting stage with the corresponding results of previous studies in Table 1. The minor differences between the present and previous volumes and ICP values can be attributable to minor clinical differences between the cases.

**Table 1:** Comparison of the results of the present study with the results of cine-PC MRI, experimental ICP, and previous studies. CSF volume means the volumes of SAS and ventricular system.

Parameters	Condition of subjects	Present study						Literature	
		FSI simulation		Experimental measurements		cine-PC MRI results			
		Value	Source	Value	Source	Value	Source	Value	Source
Maximum CSF velocity in cerebral aqueduct (cm/s)	Healthy subjects	2.3 ± 0.1	Supplementary Fig. 1	—	—	2.36	Fig. 1f	2.4	^48^
								1.3	^76^
	Communicating hydrocephalus patients (pre-shunting)	4.6 ± 0.4	Supplementary Data 1	—	—	4.7	Fig. 1f	4.1	^48^
								3.5	^76^
CSF volume (ml)	Healthy subjects	116.3 ± 1.6	Supplementary Fig. 2	—	—	—	—	129.3	^48^
								118.6	^76^
	Communicating hydrocephalus patients (pre-shunting)	412.8 ± 6.0	Fig. 2a and Supplementary Data 1	—	—	—	—	410.2	^48^
								371.1	^76^
Brain volume (ml)	Healthy subjects	1213.3 ± 10.3	Supplementary Fig. 3	—	—	—	—	1388.0	^48^
	Communicating hydrocephalus patients (pre-shunting)	1068.0 ± 19.8	Fig. 2b and Supplementary Data 1	—	—	—	—	1120.3	^48^
Maximum ICP (cm H_2_O)	Healthy subjects	10.3 ± 0.2	Supplementary Fig. 4	—	—	—	—	>9.5	^94^
	Communicating hydrocephalus patients (pre-shunting)	29.5 ± 0.7	Fig. 2c and Supplementary Data 1	28.9	Fig. 1f and Supplementary Table 1	—	—	27.5	^47^
								26.8	^48^
								29.5	^76^

### Changes in effective parameters over 15 months after shunting

**Fig. 2:**
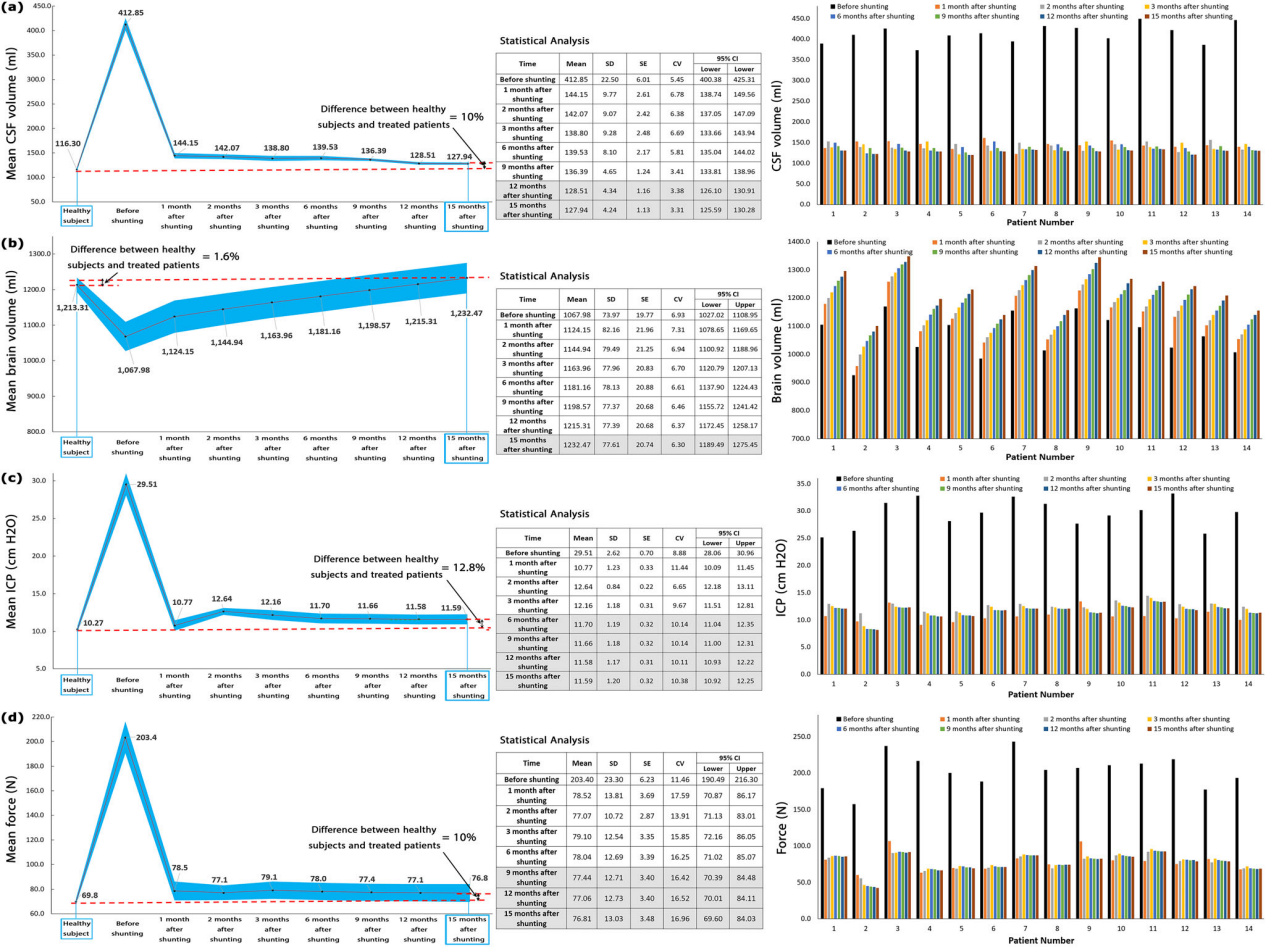
Changes in volumes of CSF and brain, ICP, and force after shunting. **a** Mean CSF volume and CSF volumes of all patients before shunting until 15 months after shunting. **b** Mean brain volume and brain volumes of all patients before shunting until 15 months after shunting. **c** Mean ICP and ICPs of all patients before shunting until 15 months after shunting. **d** Mean force and forces in all patients before shunting until 15 months after shunting. It should be noted that the mean volumes, ICP, and force for healthy subjects also showed on the left side panels. The gray areas in statistical analysis tables show the stable areas of volumes, ICP, and fore changes. The CSF volume, brain volume, ICP, and forces values reached stable conditions 12, 15, 6, and 9 months after shunting, respectively. Since the differences between the values of these parameters in stable months were less than 1.0%. Hence, follow-up was stopped 15 months after shunting, and results were reported until the fifteenth month. Raw data for Fig. 2 is included in Supplementary Data 1. CSF cerebrospinal fluid, ICP intracranial pressure, SD standard deviation, SE standard error, CV coefficient of variation, CI confidence of interval.

CSF and brain volumes in patients with successful shunting were accompanied by a 69.0% decrease and a 15.4% increase in the fifteen months, respectively (Fig. 2a, b, and Supplementary Data 1). The volume change of CSF in subarachnoid space (SAS) is negligible^17,45^, hence, the increase in CSF volume is caused by a considerable change in the size of the ventricular system. The results of Fig. 2c, d showed that successful shunting can lead to a 60.7% and 62.2% decrease in ICP and force values, respectively, 15 months after shunting.

### Volumetric strain and creep

**Fig. 3:**
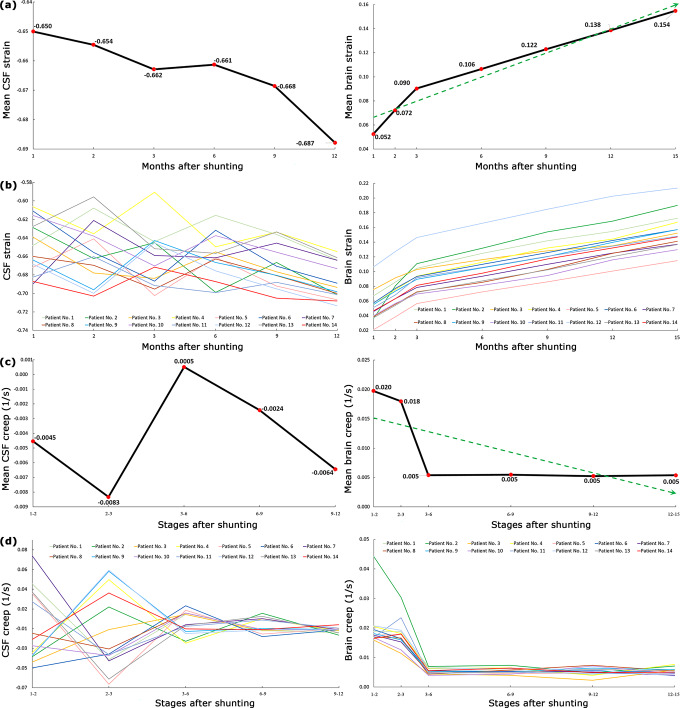
Changes in strain and creep of CSF and brain after shunting. **a** Mean CSF and brain strain. **b** CSF and brain strain of all patients until 15 after shunting. **c** Mean CSF and brain creep. **d** CSF and brain creep of all patients until 15 after shunting. CSF volume changes after the twelfth month were negligible, hence, CSF strain and creep values after the twelfth month were not calculated. The reason for the negative values of CSF strain is the descending trend of CSF volume changes after shunting. “Stages 1–2” means the CSF volumetric creep between the first and second months after shunting. Raw data for Fig. 3 is included in Supplementary Tables 2–5. CSF Cerebrospinal fluid.

We calculated the 3-dimensional strain, (V-Vo)/Vo, for the CSF and brain after shunting (Fig. 3a, b). During the reloading process following shunting, the CSF and brain volumes prior to shunting were considered as initial volumes (Vo) of CSF and brain in the strain formula. The results showed that the mean CSF volumetric strain 1 and 12 months after shunting were −0.65 ± 0.008 and −0.69 ± 0.005, respectively (Fig. 3a and Supplementary Table 2). Defining and understanding the concept of strain and creep for CSF which can be drained by shunt continually is a little challenging. The mean brain volumetric strain 1 and 15 months after shunting was 0.05 ± 0.006 and 0.15 ± 0.007, respectively (Fig. 3a and Supplementary Table 3). Contrary to CSF, the brain volumetric strain had considerable changes, and at the end of the fifteenth month, it was 3 times that of the first month after shunting. The results of Fig. 3a and Supplementary Tables 2, and 3 showed that the volumetric residual strain of CSF and brain (after 15 months) were −0.69 ± 0.005 and 0.15 ± 0.007, respectively. The slopes of volumetric strain-time curves in Fig. 3a, b are the volumetric creeps of CSF and the brain that are shown in Fig. 3c, d. The results of Fig. 3c and Supplementary Table 4 showed that mean CSF volumetric creep 1–2 and 9–12 months after shunting were −0.004 ± 0.010 s^−1^ and −0.006 ± 0.001 s^−1^, respectively. The mean brain volumetric creep 1–2 and 12–15 months after shunting were 0.020 ± 0.002 s^−1^ and 0.005 ± 0.000 s^−1^, respectively (Fig. 3c and Supplementary Table 5).

### Brain stiffness

**Fig. 4:**
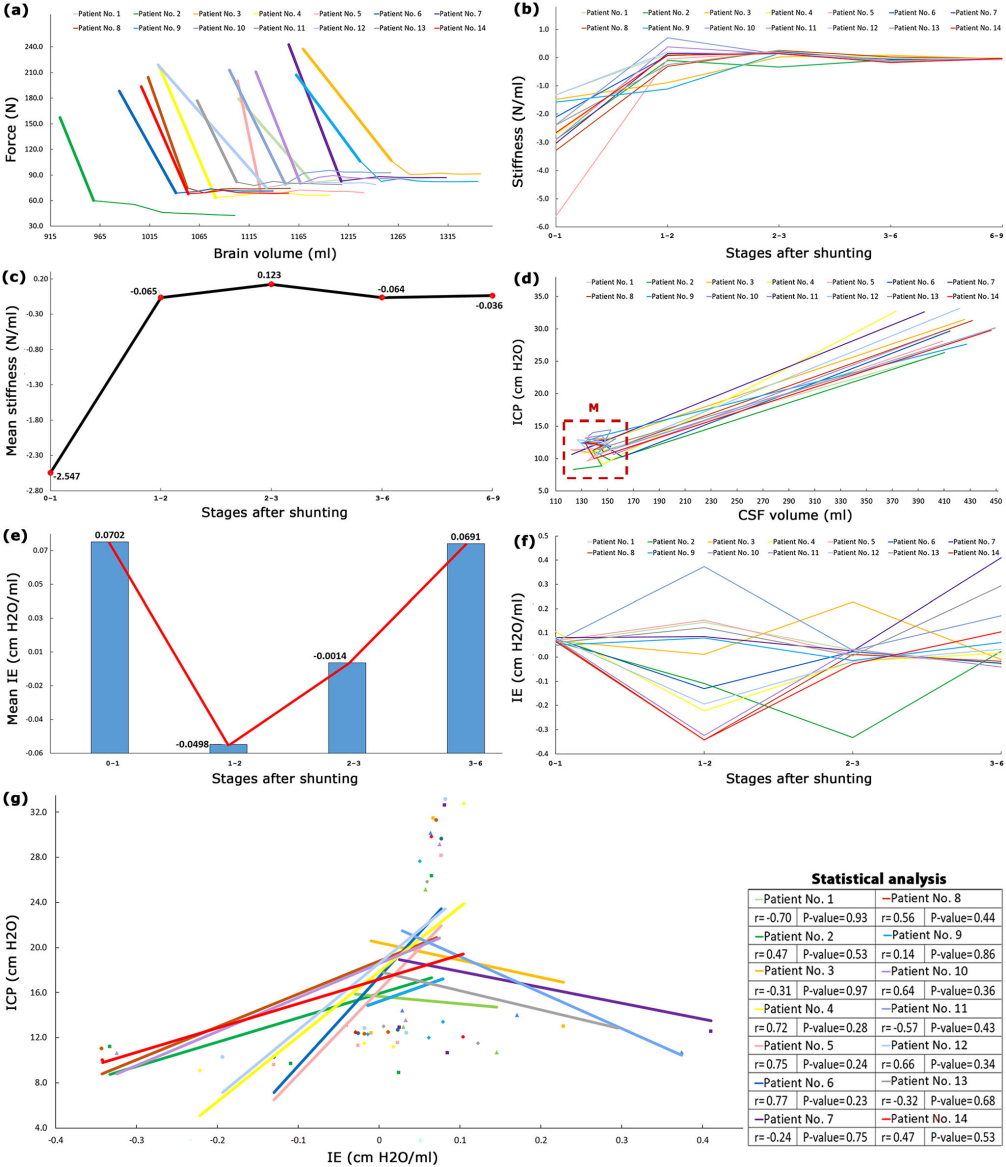
Changes in brain stiffness and elastance after shunting. **a** shows the changes in the force exerted by CSF on the brain material versus brain volume. **b** and **c** show the stiffness and mean stiffness changes in hydrocephalic brains after shunting, respectively. Force changes after the ninth month were negligible, hence, body force versus brain volume and consequently stiffness after the ninth month were not calculated in (**b**) and (**c**). **d** changes of ICP versus CSF volume for all patients after shunting. **e** and **f** are mean IE and IEs of all patients after shunting, respectively. ICP changes after the sixth month were negligible, hence, IE values after the sixth month were not calculated. “Stages 0–1” means the IE is calculated between before shunting to 1 month after shunting. **g** shows the results of the Pearson correlation coefficient for assessing the relationship between ICP and CSF volumes in all patients. P-values for all patients were higher than 0.23 and non-significant. Raw data for Fig. 4a–f are included in Supplementary Data 1, Supplementary Table 6, Supplementary Table 6, Supplementary Data 1, Supplementary Table 7, and Supplementary Table 7, respectively. Raw data for Fig. 4g is included in Supplementary Table 7 and Supplementary Data 1. CSF cerebrospinal fluid, ICP intracranial pressure, IE intracranial elastance.

The vertical axis of Fig. 4a is the distributed body force exerted by CSF on the brain tissue. The horizontal axis is brain volume that can be representative of the 3-D deformation of the brain. The slopes of the curves in Fig. 4a are representative of the hydrocephalic brain stiffness^54,55^. The results of Fig. 4b, c, and Supplementary Table 6 showed the changes in hydrocephalic brain stiffness after shunting. The bold lines in Fig. 4a showed that maximum stiffness (highest slope) was −2.547 ± 0.3 N/ml. The variations in the mean stiffness of the hydrocephalic brain after shunting are shown in Fig. 4c: −2.547, −0.065, 0.123, −0.064, and −0.036 N/ml. It should be noted that the negative values of stiffness only show the directions.

### Intracranial elastance

The slopes of ICP-CSF volume curves in Fig. 4d showed IE (ΔICP/ΔCSF volume) over an extended period of time after shunting (long-IE). Based on the definition of slope (derivative) in mathematical science, we compared the ICP and CSF volume of each stage compared to its previous step to calculate long-IE. After shunting, the changes in mean values of long-IE were oscillatory: 0.0702, −0.0498, −0.0014, and 0.0691 cm H_2_O/ml (Fig. 4e and Supplementary Table 7). Some negative slopes in the ICP-CSF volume graphs (and consequently negative IE) are shown in Fig. 4d–f. These negative slopes were also observed in a study by Okon et al.^56^ in a short-time assessment.

## Discussion

**Fig. 5:**
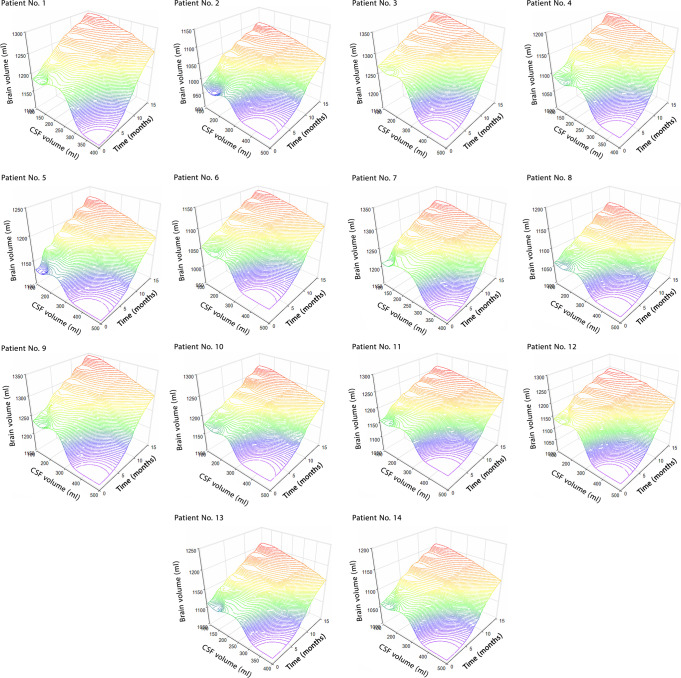
Changes in ICP, CSF volume, and time concurrently. The changes in CSF volume and brain volume with respect to time (months after shunting). The plans show the concurrent changes in volumes after shunting. Raw data for Fig. 5 is included in Supplementary Table. CSF cerebrospinal fluid.

Complex biomechanical recovery behavior of the hydrocephalic brain after shunting may an important reason for unpredictable complexities in shunt outcomes^57–60^. Hence, we performed a non-invasive computer simulation to calculate the volumes of CSF and brain, ICP, and exerted force on the brain tissue of hydrocephalus patients over 15 months after shunting. Based on these results, we evaluated the recovery behavior of the hydrocephalic brain over an extended period of time after shunting. Figure 5 shows the descending and ascending trends of CSF and brain volume changes concurrently with respect to time after shunting. The patients’ information before contracting hydrocephalus was not available. Hence, the mean values of CSF volume, brain volume, ICP, and force of healthy subjects were considered representative of similar parameters in the patients before contracting hydrocephalus (normal condition). The mean CSF volume, ICP, and force values of treated patients were 10.0%, 12.8%, and 10.0%, respectively, higher than those of the normal condition (Fig. 2a, c, d, and Supplementary Figs. 3–5). The difference in the mean brain volume of treated patients and healthy subjects was not considerable (1.6%) (Fig. 2b and Supplementary Fig. 4). Interestingly even 15 months after shunting and after disappearing all clinical signs and symptoms of hydrocephalus, except for the brain volume, other parameters did not return to the normal range. Previous studies showed that the most common reasons for shunt failure are obstruction, infection, and overdrainage^61^. In some patients, signs, symptoms, and pain will return after shunting and this leads to an unsuccessful shunting. Successful shunting is a life-saving procedure, returning the patient to normal life. Previous studies showed the effect of supplementary tests in increasing the improvement following shunting and decision-making about shunt function^62^. Medical image is another tool that can be helpful in preventing sudden shunt failure and can give information on how a shunt is functioning. We propose that intermittent MRI can potentially give information on shunt progress.

The experimental methods for the evaluation of recovery behavior of the hydrocephalic brain are accompanied by many limitations in applying a pulsatile distributed body loading on all sides of brain tissue, making a large deformation on a material with a high depth like brain tissue, measuring brain force-deformation curve three-dimensionally, and applying the correct strain rate of hydrocephalic loading similar to actual physiological condition. Time can play a prominent role in the evaluation of the recovery behavior of hydrocephalic brains because of the viscous (time-dependent) component in brain material. The time that the brain material properties were under evaluation (loading time on brain tissue) in previous non-hydrocephalic studies was between 0.1 sec to 5 min^29,38^. Shulyakov et al observed that the changes in material properties of healthy rats’ brains did not reach a stable condition even two months after loading and two months was not enough follow-up time for brain evaluation^41^. A long-time follow-up (15 months) of actual hydrocephalus patients in the present study shows that the required times for CSF volume (12 months), brain volume (15 months), ICP (6 months), and force (9 months) to reach stable conditions are really considerable (Fig. 2).

Figure 4a–c showed that maximum stiffness occurred in stages 0–1 (pre-shunting to 1 month after shunting). In a study by Weickenmeier et al, bovine brain stiffness under a short-term 1-D force was also slightly greater in the early stages of reloading conditions^54^. There were variations and non-uniformities in hydrocephalic brain stiffness values after shunting and these variations were more considerable for CSF strain and creep values (Fig. 3). On the other hand, the mean strain and creep of the brain tissue changed monotonic and uniform, and their changes were more considerable than CSF (Fig. 3). The change in rat brain creep in a study by Wang et al under a short-time loading was also monotonic^63^.

From the biofluid approach, shunting affects the fluid movement between the extracellular space, the CSF space, and the interstitial space. The consolidation of the brain defines due to the migration of CSF through the extracellular and a minor contribution of transcellular porous medium of the brain. One of the limitations of experimental investigations in the consolidation study of brain tissue is considering the effect of the CSF absorption mechanism^64^, i.e., in a study by Franceschini et al^65^, and the present study can cover this drawback. Feng et al defined consolidation phases based on the deformation-time curve for a non-biological material^66^. The present study has also defined three consolidation phases using the brain volume-time curves of hydrocephalus patients over 15 months after shunting. *First phase:* From before shunting to one month after shunting (stages 0–1) can be defined as the first phase since a considerable brain volume increase was observed in this phase (Fig. 2b). This can be a result of considerable force reduction (61.4%) in stages 0–1 (Fig. 2d). *Second phase:* After the first month, between 2–15 months after shunting, the brain expands at a semi-constant low force (Fig. 4a). Figure 2 shows that the ICP dropped, followed by the subsequent drop in force. ICP reached the plateau condition after 6 months and the force then reached the plateau condition after 9 months with a 3-month delay. This can be related to the effect of the viscous damping component of the brain tissue. From a biomedical engineering point of view, the viscous component acts with a delay. Figures 2 and 4a confirmed this behavior and show a lag between ICP/force stable conditions and the much longer time required for CSF/brain volume to stabilize. Hence, it can be deduced that the effect of the viscous component was stronger in the second phase relative to the first phase. Because the increases in brain volume have been less in the second phase (Fig. 2b). It should be noted that in the second phase, the changes in brain volume had a totally monotonic (ascending) trend with lower slopes (Fig. 2b). *Third phase:* The viscous component overcame and damped the input load in the third consolidation phase (after the fifteenth month) and changes in brain volume were stopped.

IE is one of the most important indexes that can reflect the clinical condition of hydrocephalus patients^67^. Previous IE measurements in a short time interval (short-IE) believed that short-IE will always decrease uniformly after shunting^68^ and it is separable into two different areas: higher IE and lower IE. However, the results of the present non-invasive calculations showed that long-IE was not separable into some different areas in any of the patients since long-IE changes in all patients were non-monotonic and oscillatory (Fig. 4e, f). It should be noted that previous studies believed elastance is a linear function of ICP and there is a linear relationship between short-IE and ICP^69^. However, the results of our study in Fig. 4g showed that the relationship between long-IE and ICP is not linear in any patients (P-values>0.23). The lack of a linear relationship between long-IE and ICP may justify the lack of elevated ICP in normal pressure hydrocephalus (NPH) patients despite changes in IE. It should be noted that our system for long-IE calculation was a closed system because our patients did not experience any changes in valve adjustment.

We only evaluated the hydrocephalic brain after shunting (during reloading condition). The material properties of brain in compression (loading condition) and tension (reloading condition) are not the same^70^. It may be deduced that the force-brain volume curves and consequently brain stiffness changes during a hydrocephalic episode (compression loading condition: when a healthy subject was contracting hydrocephalus) were likely not symmetric with our corresponding curves in Fig. 4a–c. However, it needs more assessments in future studies. Comparison of the strain and creep of patients with cerebral atrophy with our similar results may be useful to prevent the misdiagnosing of some types of hydrocephalus like NPH and patients with cerebral atrophy such as Alzheimer’s and Parkinson’s. In addition, we defined the consolidation phases for the hydrocephalic brain, it is also suggested for future studies to calculate the consolidation ratios after shunting based on Terzaghi theory^65,71^. Previous studies showed some subsets of pediatric hydrocephalus may arise from genetic dysregulation of brain development, leading to the impaired biomechanical stability of the brain that facilitates secondary ventricular dilation in the absence of abnormal CSF flow^72,73^. Improvement after shunting can be related to the age and gender of patients. The present study focused on patients aged 50–72 years (Supplementary Data 1) and it would be interesting to work on younger patients in future studies. We selected 14 of 38 patients who had improved outcomes after shunting. However, changes in brain biomechanical parameters in patients with shunt revision or patients with headaches and pain without shunt revisions can be of great importance to study in future studies. Also, in future work, it will be important to examine shunt outcomes in the presence of comorbidities such as heart or abdominal disease. In addition, clinical conditions of patients before and after shunting may have a relationship with intracranial physiology and changes in the brain biomechanical parameters such as IE and stiffness, and extending this study to assess these effects can be useful for future studies.

We evaluated the brain recovery behavior of adult communicating hydrocephalus patients non-invasively over 15 months after shunting. Previous studies had shown the short-term effect of the viscous component in brain consolidation study. The present study confirms the role of the viscous component of brain tissue even in the third consolidation phase. The evaluation of the changes in strains, creeps, brain stiffness, and long-IE for assessment of brain recovery behavior in a natural and actual condition of hydrocephalus patients over an extended period of time after shunting may be helpful to improve our knowledge regarding the reason and mechanism for shunt failure.

## Materials and methods

### Research populations and magnetic resonance imaging

14 of 38 adult communicating hydrocephalus patients did not have shunt failure and the neurosurgeons did not also need to change the adjustment of the shunt valve during the 15 months. With regard to the goal of the present study, the data of these 14 patients with successful shunting without any changes in valve adjustment were used in the present study. These patients did not have any history of other central nervous system disorders and/or surgeries. For the treatment of all patients, a Medtronic ventriculoperitoneal shunt with the ventricular frontal entry site (one-way) was used. We also recruited 16 healthy subjects to compare the deviation of the hydrocephalic condition from the normal condition. Body mass index and age of patients were 24.1–28.7 kg/m^2^ and 50–72 years, respectively (Supplementary Data 1). The corresponding values for healthy subjects were 22.6–27.3 kg/m^2^ and 44–67 years, respectively. Cine-PC MRI for the head of 16 healthy subjects (eight males and eight females) was provided. This data for 14 hydrocephalus patients (seven males and seven females) were also provided in 8 stages: 0, 1, 2, 3, 6, 9, 12, and 15 months after shunting. It should be noted that 0 months after shunting means before shunting. The five most common pre-shunting signs and symptoms of patients included headache (13 patients), nausea and vomiting (7 patients), dizziness and lethargy (7 patients), sleepiness (5 patients), and cognitive difficulties (4 patients). The process of study design, protocols, and performing procedures were accepted and approved by the ethical committee of Tarish Hospital, and the 1964 Helsinki declaration and its later amendments. The data of patients and healthy subjects were anonymized and written consent was provided by all of them before starting the project.

MRI includes a cardiac-gated PC to quantify CSF velocity and an axial T2 weighted image (T2WI). The time to echo/repetition time, field of view, slice intervals/slice thickness, and flip angle for axial T2WI equaled 117/4,000 ms, 220 × 220 mm, 1.8/6 mm, and 90°, respectively. The corresponding values for PC-MRI equaled 7/21 ms, 160 × 160 mm, 1.2/6 mm, and 10°, respectively. The acquisition times for axial T2WI and PC-MRI were 150 sec and 270 sec, respectively. It should be noted that velocity encoding in PC-MRI was 15 cm/s. A 3 Tesla (Magnetom Trio, Siemens, Erlangen, Germany) was used for generating MRI data. More information about detailed MRI protocol and setting are described in the study by Long et al and Kapsalaki et al^74,75^.

The geometrical modeling process, methodology, and formulations, as well as simulation parameters including assumptions, constant values, and boundary conditions which were used in this study, are similar to a recent study done by the authors^45^. Briefly, head MRIs of hydrocephalus patients for 8 stages and healthy subjects were generated. MRI images of these 128 scannings (in DICOM extension) were transferred to Mimics software (version 15; Tissueise, Leuven, Belgium) to provide the separated points clouds of the head substructure. It should be mentioned the segmentation procedure of the fluid model (ventricular system and SAS) and solid model (brain tissue) was carried out manually in the Mimics software. This procedure was also common in previous studies for providing points clouds and consequently volume measurements^47–49,76,77^. The points clouds were transferred to SolidWorks software (version 2018; Dassault Systemes SolidWorks Corp., Waltham, MA, USA) and three-dimensional geometrical models of each patient in all stages and each healthy subject were created (Fig. 1a). The volumes of the ventricular system, SAS, and brain were measured in this step. Finally, these models were transferred to ADINA software (version 8.3; Adina R&D Inc., Watertown MA, USA) for meshing (Fig. 1b, c) and FSI simulations to calculate ICP and force excreted on the brain tissue (Fig. 1d). All the abovementioned processes were performed for 14 patients (at 8 stages) and 16 healthy subjects. It is worth mentioning that the patients’ information during contracting hydrocephalus (during the loading process) was not available. Therefore, all simulated parameters in the present study were calculated during the “reloading” process (CSF drainage due to shunting and consequently removing the compression loading on the brain material).

**Fig. 6:**
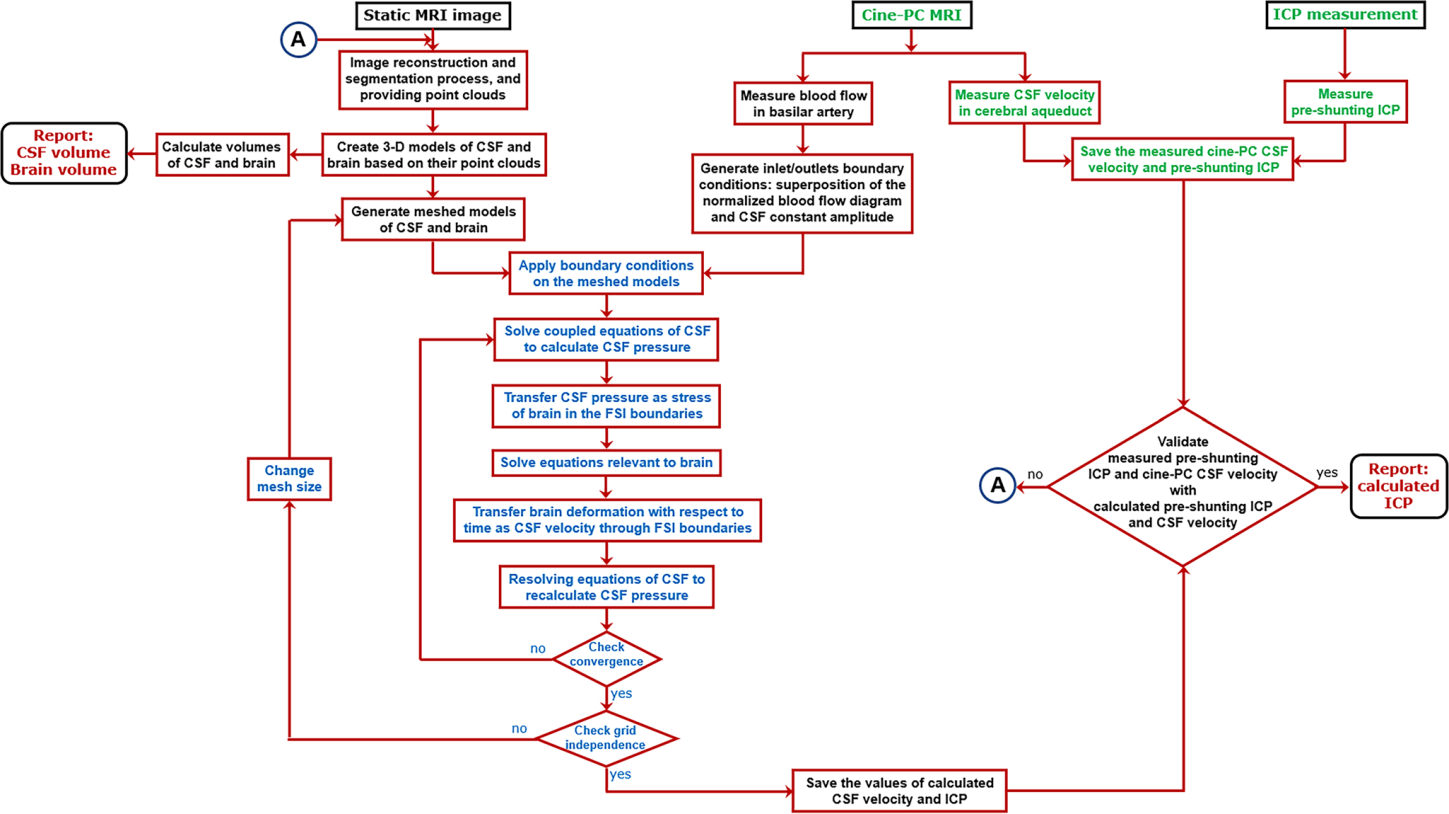
Methodological workflow. The workflow included FSI method (blue text), measured parameters (green text), and calculated parameters (maroon text).

In addition to the use of MRI data to create three-dimensional geometrical models of head substructure, these data were also used for two other goals. 128 CSF velocities in the cerebral aqueduct (measured by cine-PC MRI) were used for data validation (second MRI output). Also, blood flow in basilar arteries of all patients and healthy subjects were measured using cine-PC MRI (third MRI output) and were used to define the oscillatory component of the inlet/outlets boundary conditions during FSI simulation. The measured parameters include CSF velocities and pre-shunting ICP. The calculated parameters were CSF velocities, pre-shunting ICP, post-shunting ICP, and volumes of the brain and CSF. We used the measured and calculated CSF velocities and pre-shunting ICP for data validation. It should be noted that force, strain, creep, stiffness, and IE were calculated based on calculated volumes and ICP values which were validated with measured data. The FSI method, measured parameters, and calculated parameters are shown in blue text, green text, and maroon text, respectively in Fig. 6. It should be noted that strain shows the volume change of intracranial components under different pressures. Shunting leads to a pressure change, and strain is representative to show the volume change of the intracranial component. When the volume of intracranial components changes as a result of shunting, creep indicates if this volume change will occur slowly or quickly. Elastance represents the resistance of the brain to volume change under changes in ICP.

### Fluid-structure interaction formulation

Non-invasive measurement of the effective parameters in brain evaluation is of great importance. Force exerted on the brain tissue and ICP are not measurable using non-invasive and/or in vivo methods. Invasive tools can be applied to measure the force at a specific point of the brain tissue, not a 3-D distributed body force similar to actual physiological conditions of hydrocephalus patients^21,41^. Hence, computer simulation can be a non-invasive option to calculate force and ICP. Computational fluid dynamics (CFD) and FSI are to most common methods for simulation of biological phenomena^78^. With regard to the results of our recent study, the FSI method can lead to a more accurate result to simulate hydrocephalic condition^45^, since the interfaces between fluid and solid models are deformable. Alperin et al and Mase et al tried to calculate ICP non-invasively using the continuity and Navier-Stokes equations^79–81^. Our recent study identified the details of optimal parameters in FSI simulation for the robust and reliable evaluation of hydrocephalus patients non-invasively^45^. Therefore, we have used this optimized FSI method in the present study. A coupled 2-way FSI based on arbitrary Lagrangian-Eulerian formulations was used in the present study to calculate force and ICP for healthy subjects and hydrocephalus patients in all 8 stages. The governing equations of CSF were the coupled equations of continuity and Navier-Stokes. Equations (1) and (2) reflect the continuity equations for the ventricular system and SAS, respectively. The Navier-Stokes and solid equations are given by Eqs. (3) and (4), respectively^17,43,48,82,83^. It should be noted that the first node of elements is defined as the reference point for software calculations^84–86^.

1 $$\nabla . {{{{{{\boldsymbol{u}}}}}}}_{F} \, = \, S$$

2 $$\nabla . {{{{{{\boldsymbol{u}}}}}}}_{F} \, = \, 0$$

3 $${\rho }_{F}\frac{\partial {{{{{{\boldsymbol{u}}}}}}}_{{{{{{\boldsymbol{F}}}}}}}}{\partial t}+{\rho }_{F}\left(\left({{{{{{\boldsymbol{u}}}}}}}_{F}-{{{{{\boldsymbol{W}}}}}}\right) . \nabla \right){{{{{{\boldsymbol{u}}}}}}}_{F} \, = \, - \nabla {{{{{\rm{p}}}}}} \, + \, \mu {\nabla }^{{{{{{\bf{2}}}}}}}{{{{{{\boldsymbol{u}}}}}}}_{F}+{{{{{{\boldsymbol{f}}}}}}}_{F}^{{{{{{\boldsymbol{B}}}}}}}$$

4 $$\nabla . {{{{{{\boldsymbol{\sigma }}}}}}}_{{{{{{\boldsymbol{S}}}}}}}+{{{{{{\boldsymbol{f}}}}}}}_{F}^{{{{{{\boldsymbol{B}}}}}}} \, = \, {\rho }_{S}\ddot{{{{{{{\boldsymbol{u}}}}}}}_{S}}$$

where (u_F_-W) is a representative for the relative velocity vector of fluid (CSF) concerning the mobile coordinate velocity. u_F_ and W can be defined separately as velocities of CSF and moving mesh, respectively. Brain and CSF densities are shown by ρ_S_ and ρ_F_, respectively. CSF pressure and viscosity (dynamic) are represented by P and µ, respectively. S is a representative of the CSF production in lateral ventricles. The body force is also defined per unit volume that is shown by ${f}_{F}^{B}$. In the governing equation of the brain (Eq. 4), $\ddot{{u}_{S}}$ and *σ*_*S*_ are local acceleration and stress of the brain, respectively. Material properties of CSF as an incompressible Newtonian fluid includes: µ = 0.001 kg.m^−1^.s^−1^ and ρ_F_ = 998.2 kg/m^3^ ^17,43,45,50^.

### Constitutive model and boundary conditions

The role of a time-dependent parameter (viscous component) in brain material is undeniable^87,88^. Some previous studies have used viscoelastic, poroelastic, and hyper-viscoelastic as constitutive models for brain material of hydrocephalus patients^43,47–50,89^. However, it has been proven that poro-viscoelastic can be the most accurate constitutive model to define material properties of brain tissue for hydrocephalus patients because of higher conformity with the empirical findings^17,45,88,90^.

Total equations were obtained based on the brain equation, Darcy equation for the porous medium, and stress equilibrium based on the study by Gholampour et al and Cheng et al^17,45,90,91^. Elkin et al compared some models to fit the viscoelastic component of the brain tissue^92^. Their results showed that the Prony series is an accurate option to define the viscoelasticity of the brain tissue. Therefore in the present study, the viscoelastic component of the solid model (brain) is defined through the Prony series^17,90^. The equation of shear relaxation modulus that depends on time is shown in Eq. 5^ 17,90^.

5 $${{{{{\rm{Gr}}}}}}(t)={{{{{{\rm{G}}}}}}}_{0}\left(1-\mathop{\sum }\limits_{k=1}^{N}{g}_{k}^{p}\left({1-e}^-\left({\frac{t}{{\tau }_{k}}}\right)\right)\right)$$

In Eq. 5, the instantaneous shear modulus is shown by G_0_. ${g}_{k}^{p}$, *τ*_1_, *τ*_2_, and *τ*_3_ are defined as equal 0.285, 3.1, 27, and 410, respectively^17,50,90,91^. Young modulus and the Poisson ratio of brain tissue are defined as equal to 584.4 Pa and 0.35, respectively^45^. The permeability and void ratio of the brain tissue are considered 4.08 × 10^−12^ m^4^/N.s and 0.2, respectively^17,50,90^. It is worth mentioning that the correctness of these constant parameters to simulate the hydrocephalic brain was validated in previous studies^17,50,90,91^.

The majority of previous brain simulations were carried out to calculate small-deformations while hydrocephalus usually occurs with large-deformation. The most important concern to simulate based on large-deformation is boundary conditions^93^. On the other hand, FSI simulation is also very sensitive to the correct boundary conditions^45^. Recently we compared all existing boundary conditions for hydrocephalus simulation and we revealed CSF pulsatile flow rates are the optimal inlet/outlets boundary conditions with appropriate conformity with the physiological condition of hydrocephalus patients^45^. Despite various sources for CSF production, the lateral ventricles were defined as the location of inlet CSF flow in FSI simulations similar to previous studies^17,43,45,48,50^. Because the majority of CSF is produced in the lateral ventricle. Similarly, despite various sources for CSF absorption such as arachnoid granulations, intraparenchymal routes, and extra-cranial lymphatic pathways, we defined merely two main outlets for CSF flow similar to previous studies^17,45^: sagittal sinus and spinal cord. To calculate one inlet CSF flow and two outlets CSF flow, we superposed a constant amplitude with a pulsatile function^45^ in MATLAB software (version R2018; Mathworks, Natick, MA, USA). The constant amplitudes were obtained based on the physiological values of CSF production: inlet CSF flow rate = 0.35 ml/min, outlet CSF flow rate in sagittal sinus = 0.18 ml/min, and outlet CSF flow rate in spinal cord = 0.17 ml/min^45^. The pulsatile functions for inlet and outlets were accessed through the normalization of the blood flow rate diagram in the basilar arteries (third MRI output). Using the normalization of the blood flow rate-time diagram, we obtained the pulsatile behavior independent of magnitudes, as the maximum and minimum values of the normalization diagrams were 1 and −1, respectively^45^. It is worth mentioning that for all 128 simulations (14 patients in 8 stages and 16 healthy subjects), these procedures were performed by MATLAB, and these data were transferred to ADINA as inlet/outlets boundary conditions. With regard to our recent study that showed that the effect of the outer layer of the SAS (dura mater and skull) on hydrocephalus simulation of adult patients is negligible^45^, the dura mater and skull were not considered in our simulations, and 3-D geometrical models included solid domain (brain) and fluid domain (SAS and ventricular system) (Fig. 1c). Hence, except for outer layer of SAS that defined by a no-slip boundary condition, other layers (outer and inner layer of brain that contacted with CSF) were defined as FSI boundary conditions (Fig. 1c).

Equation 6 can describe the displacement compatibility in the FSI boundaries between CSF and brain models^17,43^. With regard to Eqs. 7 and 8, the traction forces and velocities of CSF and brain domains have to be equaled in the FSI interface^17,43^.

6 $${{{{{{\boldsymbol{d}}}}}}}_{S}={{{{{{\boldsymbol{d}}}}}}}_{F}(x,y,z)\epsilon {\varGamma }_{{wall}}^{F}\cap {\varGamma }_{{wall}}^{S}$$

7 $${{{{{{\boldsymbol{\sigma }}}}}}}_{S}.{{{{{\boldsymbol{n}}}}}}={{{{{{\boldsymbol{\sigma }}}}}}}_{F}.{{{{{\boldsymbol{n}}}}}}(x,y,z)\epsilon {\varGamma }_{{wall}}^{F}\cap {\varGamma }_{{wall}}^{S}$$

8 $${{{{{{\boldsymbol{u}}}}}}}_{S}={{{{{{\boldsymbol{u}}}}}}}_{F}(x,y,z)\epsilon {\varGamma }_{{wall}}^{F}\cap {\varGamma }_{{wall}}^{S}$$

where d_S_ and d_F_ are displacements of brain and CSF in FSI boundaries, respectively. u_s_ is the brain displacement in FSI boundary with respect to time. The stress tensors for brain and CSF in FSI interfaces are defined as *σ*_*S*_.*n* and *σ*_*F*_.*n*, respectively.

It should be noted that we added a shunt tube on the right lateral ventricle in the patients’ models after shunting (at 7 stages). The inner diameter of the tube was 1.3 mm. We did not change the outlet boundary condition of the tube during the simulation process. Because we only used patients without any changes in valve adjustment during 15 months. We defined a pressure diagram as the outlet boundary condition of the shunt tube^17^. The minimum and maximum pressure were defined as 14.5 cm H_2_O and 17.0 cm H_2_O, respectively, similar to adjusted valve pressure in patients. Detailed information for tube inner diameter and pressure outlet values are available in the Medtronic catalog: Medtronic, Strata Various Adjustment System, Minnesota, USA.

It should be mentioned that the maximum CSF pressure value in SAS specifically in the upper convexity of the brain defined as ICP in the present study. The force can be calculated in any direction or location using ADINA software. We calculated and reported the maximum global force exerted on the brain tissue of patients (at 8 stages), and healthy subjects.

### Grid independence study

Tetrahedral four-node meshes were used to mesh generation of the fluid and solid models in ADINA software. The results of the grid independence study showed that the differences between very fine mesh and fine mesh in all simulations were less than 0.36% (Fig. 1e). The numbers of fine and very fine meshes for healthy subject No. 1 were 985,238 and 1,311,594, respectively. The corresponding values for patient No. 1 before shunting were 1,216,850 and 1,816,024, respectively. The comparable values for this patient 15 months after shunting were 1,106,470 and 1,524,407, respectively. It should be noted that the time step was 0.01 in all simulations. To refine the grids of meshes, the implicit Euler scheme was used. Decreasing the step sizes did not lead to differences in the force and ICP values.

After the grid independence check, the results showed that there were not any differences between force and ICP diagrams in the fourth and fifth cardiac cycles. In the present study, the force and ICP values in the fourth cardiac cycle were reported for healthy subjects and patients in all stages.

### Statistics and reproducibility

The percentages of variables for comparing the changes of each parameter in different conditions were calculated. The mean, standard deviation (SD), and standard error (SE) were calculated for each volume, force, and ICP dataset using IBM SPSS software, version 20.0, IBM Corp, Armonk, NY, USA (Supplementary Tables 1–7, Supplementary Data 1, Supplementary Figs. 1–6, and Fig. 2). In addition, coefficient of variation (CV) and confidence interval (CI) were also calculated for all datasets (Supplementary Tables 1–7, Supplementary Figs. 1–6, and Fig. 2). The results of Shapiro-Wilk tests confirmed the normal distributions in all datasets. The parametric ANOVA multiple comparisons were performed using IBM SPSS software to compare all data in the various conditions and different stages after shunting as well as between treated patients (15 months after shunting) and healthy subjects. The homogeneity evaluation of the variance test showed equal variances. To compare the datasets after ANOVA, Tukey’s posthoc test was performed for pairwise comparison. We also used Student’s t-test to compare the results of CINE PC-MRI and FSI simulation for CSF velocity in the cerebral aqueduct. T and F were the test statistics for Student’s t-test and ANOVA, respectively. Pearson correlation coefficient was performed to assess the relationship of intracranial elastance with ICP. In addition, all values were stated as mean ± standard error, and P-value = 0.05 was defined as the threshold for statistical significance. It is worth mentioning that to calculate the “mean” of each parameter, first, the maximum value of that pulsatile parameter was calculated in a stage. After, the average of these maximum values was calculated for all patients in that specific stage.

### Reporting summary

Further information on research design is available in the Nature Research Reporting Summary linked to this article.

## Supplementary information

Supplementary Materials

Description of Additional Supplementary Files

Supplementary Data 1

Reporting Summary

## Data Availability

All data used in this manuscript are publicly available and can be found in original publications or repositories. The MRI files of subjects, however, contain some identifying information about patients and normal subjects, and cannot be made publicly available. The data are available from the corresponding author.

## Code availability

The MATLAB codes and simulation process setting are available upon request to corresponding author.
