# Supplementary material for: Long-term recovery behavior of brain tissue in hydrocephalus patients after shunting
Source: Commun Biol. 2022 Nov 8;5:1198. doi: 10.1038/s42003-022-04128-8 (PMC9640582; doi:10.1038/s42003-022-04128-8)
Supplement: Supplementary file 1 — Supplementary Materials [file 42003_2022_4128_MOESM1_ESM.pdf]

Long-term recovery behavior of brain tissue in hydrocephalus patients after shunting

Seifollah Gholampour[1], David Frim[1], Bakhtiar Yamini[1*]

[1] Department of Neurological Surgery, University of Chicago, Chicago, Illinois, USA.

***Correspondence:** Bakhtiar Yamini, MD,

Department of Neurological Surgery

The University of Chicago

5841 S. Maryland Ave, 60637, Chicago, IL, USA.

Tel: (+1) 773-702-2123

Fax: (+1) 773-702-3518

E-mail: byamini@bsd.uchicago.edu

Short Title: Hydrocephalic brain recovery after shunting

Number of supplementary Tables: 7

Number of supplementary Figures: 6

Supplementary Table 1. Validation of the calculated CSF velocity in the cerebral aqueduct and calculated ICP.

CSF: Cerebrospinal fluid; ICP: Intracranial pressure; FSI: Fluid-structure interaction; Cine-PC MRI: Cine phase-contrast magnetic resonance imaging; SD: standard deviation; CV: coefficient of variation; CI: confidence of interval.

Comparison of maximum CSF velocity (cm/s)																					
	Healthy 1	Healthy 2	Healthy 3	Healthy 4	Healthy 5	Healthy 6	Healthy 7	Healthy 8	Healthy 9	Healthy 10	Healthy 11	Healthy 12	Healthy 13	Healthy 14	Healthy 15	Healthy 16	Mean	SD	CV	95% CI	
																				Lower	Upper
FSI simulation	1.52	2.76	2.12	1.85	2.89	3.14	1.78	2.14	2.05	2.56	1.84	3.04	2.74	1.74	2.07	2.54	2.30	0.49	21.51	2.30	2.30
Cine-PC MRI	1.57	2.84	2.06	1.79	2.91	3.12	1.83	2.20	2.05	2.48	1.91	2.95	2.75	1.74	2.01	2.62	2.30	0.49	21.14	2.30	2.30
Comparison of maximum pre-shunting CSF velocity (cm/s)																					
	Patient 1	Patient 2	Patient 3	Patient 4	Patient 5	Patient 6	Patient 7	Patient 8	Patient 9	Patient 10	Patient 11	Patient 12	Patient 13	Patient 14	---	---	Mean	SD	CV	95% CI	
																				Lower	Upper
FSI simulation	5.11	3.32	6.65	2.43	4.95	6.03	4.18	5.81	3.54	6.91	5.42	3.67	4.03	2.91	---	---	4.64	1.36	29.24	4.64	4.64
Cine-PC MRI	4.95	3.42	6.62	2.5	4.89	6.14	4.3	5.98	3.43	6.84	5.37	3.56	4.07	2.86	---	---	4.64	1.36	29.27	4.64	4.64
Comparison of maximum pre-shunting ICP (cm H2O)																					
	Patient 1	Patient 2	Patient 3	Patient 4	Patient 5	Patient 6	Patient 7	Patient 8	Patient 9	Patient 10	Patient 11	Patient 12	Patient 13	Patient 14	---	---	Mean	SD	CV	95% CI	
																				Lower	Upper
Experimental	24.3	25.4	32.8	32.1	27.1	31.0	---	---	28.7	27.9	---	32.4	24.8	31.2	---	---	28.88	3.04	10.54	28.88	28.88
FSI simulation	25.12	26.35	31.47	32.76	28.14	29.67	---	---	27.64	29.14	---	33.18	25.84	29.81	---	---	29.01	2.58	8.91	29.01	29.01

Supplementary Table 2. CSF volumetric strain of patients from before shunting to 15 months after shunting. After the twelfth month, the differences between the CSF volumes were negligible, hence, the CSF strain values are not calculated after 12 months.

CSF: cerebrospinal fluid; SD: standard deviation; SE: standard error; CV: coefficient of variation; CI: confidence of interval.

Patient No. / Months after shunting	1	2	3	4	5	6	7	8	9	10	11	12	13	14	Mean	SD	SE	CV	95% CI	
																			Lower	Upper
1	-0.648456	-0.628781	-0.639157	-0.606223	-0.671810	-0.610882	-0.690000	-0.659925	-0.663842	-0.615605	-0.682554	-0.668414	-0.627625	-0.686898	-0.6500	0.029	0.008	-4.440	-0.666	-0.634
2	-0.607853	-0.662174	-0.677920	-0.635461	-0.640997	-0.655780	-0.621107	-0.669655	-0.696147	-0.637982	-0.660293	-0.699960	-0.595515	-0.702811	-0.6545	0.034	0.009	-5.119	-0.673	-0.636
3	-0.644087	-0.645112	-0.683793	-0.590665	-0.702379	-0.686678	-0.658869	-0.695369	-0.642774	-0.670305	-0.691458	-0.645407	-0.651708	-0.671657	-0.6629	0.029	0.008	-4.448	-0.679	-0.647
6	-0.615563	-0.698735	-0.655132	-0.649678	-0.661050	-0.631641	-0.661404	-0.662705	-0.666417	-0.637485	-0.699027	-0.675530	-0.656628	-0.687346	-0.6613	0.024	0.006	-3.624	-0.675	-0.648
9	-0.636378	-0.666561	-0.676745	-0.633852	-0.692108	-0.670505	-0.645690	-0.680775	-0.680463	-0.655387	-0.687897	-0.694979	-0.633581	-0.705052	-0.6686	0.024	0.006	-3.589	-0.682	-0.655
12	-0.664131	-0.701660	-0.693424	-0.655043	-0.706781	-0.689092	-0.664191	-0.700002	-0.698254	-0.673289	-0.701253	-0.713479	-0.661548	-0.708190	-0.6879	0.020	0.005	-2.905	-0.699	-0.677

Supplementary Table 3. Brain volumetric strain of patients from before shunting to 15 months after shunting.

SD: standard deviation; SE: standard error; CV: coefficient of variation; CI: confidence of interval.

Patient No. / Months after shunting	1	2	3	4	5	6	7	8	9	10	11	12	13	14	Mean	SD	SE	CV	95% CI	
																			Lower	Upper
1	0.066983	0.036359	0.075588	0.055158	0.021061	0.057582	0.045796	0.038944	0.055344	0.040145	0.051315	0.106541	0.037413	0.046854	0.05251	0.021	0.006	39.513	0.041	0.064
2	0.085813	0.080590	0.091492	0.075649	0.038642	0.077084	0.063373	0.055821	0.073496	0.056906	0.068207	0.127255	0.053974	0.063346	0.07226	0.021	0.006	29.304	0.061	0.084
3	0.104553	0.110979	0.102864	0.093797	0.056495	0.093437	0.078612	0.072401	0.089498	0.069477	0.091673	0.146503	0.071946	0.081328	0.09025	0.022	0.006	24.627	0.078	0.103
6	0.124922	0.131851	0.116289	0.112629	0.072535	0.109791	0.093678	0.085429	0.104984	0.081512	0.105734	0.165849	0.087284	0.097920	0.10646	0.024	0.006	22.533	0.093	0.120
9	0.141941	0.154021	0.128174	0.132144	0.086038	0.125636	0.109783	0.102799	0.120383	0.094707	0.120709	0.184707	0.102339	0.117194	0.12290	0.026	0.007	20.767	0.109	0.137
12	0.154706	0.168620	0.135100	0.144145	0.100629	0.142193	0.125196	0.124808	0.139568	0.116015	0.134770	0.202490	0.120406	0.131997	0.13862	0.025	0.007	17.908	0.125	0.152
15	0.172812	0.190141	0.152458	0.167270	0.115038	0.157225	0.137145	0.141191	0.157291	0.129922	0.148192	0.213726	0.136873	0.147197	0.15475	0.025	0.007	16.366	0.141	0.169

Supplementary Table 4. CSF volumetric creep of patients from before shunting to 15 months after shunting. After the twelfth month, the differences between the CSF volumes were negligible, hence, the CSF creep values are not calculated after 12 months. Stages 1-2 means the CSF volumetric creep between the first and second months after shunting.
CSF: cerebrospinal fluid; SD: standard deviation; SE: standard error; CV: coefficient of variation; CI: confidence of interval.

Stages \ Patient No.	1	2	3	4	5	6	7	8	9	10	11	12	13	14	Mean	SD	SE	CV	95% CI	
																			Lower	Upper
1-2	0.040602	-0.033393	-0.038762	-0.029238	0.030814	-0.044898	0.068893	-0.009730	-0.032305	-0.022377	0.022261	-0.031546	0.032110	-0.015913	-0.00453	0.036	0.010	-796.543	-0.025	0.015
2-3	-0.036234	0.017062	-0.005873	0.044796	-0.061383	-0.030898	-0.037763	-0.025714	0.053373	-0.032323	-0.031166	0.054553	-0.056193	0.031153	-0.00833	0.041	0.011	-487.717	-0.031	0.014
3-6	0.009508	-0.017874	0.009554	-0.019671	0.013776	0.018346	-0.000845	0.010888	-0.007881	0.010940	-0.002523	-0.010041	-0.001640	-0.005230	0.00052	0.012	0.003	2284.957	-0.006	0.007
6-9	-0.006938	0.010725	-0.007204	0.005275	-0.010353	-0.012955	0.005238	-0.006023	-0.004682	-0.005967	0.003710	-0.006483	0.007682	-0.005902	-0.00242	0.007	0.002	-304.720	-0.007	0.002
9-12	-0.009251	-0.011700	-0.005560	-0.007064	-0.004891	-0.006196	-0.006167	-0.006409	-0.005930	-0.005967	-0.004452	-0.006167	-0.009322	-0.001046	-0.00644	0.002	0.001	-38.819	-0.008	-0.005

Supplementary Table 5. Brain volumetric creep of patients from before shunting to 15 months after shunting. The unit of all values is s^{-1}. Stages 1-2 means the CSF volumetric creep between the first and second months after shunting.

SD: standard deviation; SE: standard error; CV: coefficient of variation; CI: confidence of interval.

Patient No. / Stages	1	2	3	4	5	6	7	8	9	10	11	12	13	14	Mean	SD	SE	CV	95% CI	
																			Lower	Upper
1-2	0.018830	0.044232	0.015904	0.020490	0.017581	0.019502	0.017577	0.016877	0.018153	0.016761	0.016892	0.020714	0.016561	0.016492	0.01975	0.007	0.002	36.467	0.016	0.024
2-3	0.018740	0.030389	0.011372	0.018149	0.017853	0.016353	0.015239	0.016580	0.016002	0.012571	0.023466	0.019248	0.017972	0.017982	0.01799	0.005	0.001	25.572	0.015	0.021
3-6	0.006790	0.006957	0.004475	0.006277	0.005347	0.005451	0.005022	0.004343	0.005162	0.004012	0.004687	0.006449	0.005113	0.005530	0.00540	0.001	0.000	16.985	0.005	0.006
6-9	0.005673	0.007390	0.003962	0.006505	0.004501	0.005282	0.005368	0.005790	0.005133	0.004398	0.004991	0.006286	0.005018	0.006425	0.00548	0.001	0.000	17.111	0.005	0.006
9-12	0.004255	0.004867	0.002309	0.004001	0.004863	0.005519	0.005137	0.007336	0.006395	0.007103	0.004687	0.005928	0.006022	0.004934	0.00524	0.001	0.000	24.912	0.005	0.006
12-15	0.006035	0.007174	0.005786	0.007708	0.004803	0.005011	0.003983	0.005461	0.005907	0.004636	0.004474	0.003745	0.005489	0.005067	0.00538	0.001	0.000	20.692	0.005	0.006

Supplementary Table 6. Brain stiffness for patients from before shunting to 15 months after shunting. The unit of all values is N/ml. The force changes after the ninth month was negligible. Hence, the stiffness values measure to the ninth month. Stages 0-1 means the brain stiffness between before shunting and the first month after shunting.

SD: standard deviation; SE: standard error; CV: coefficient of variation; CI: confidence of interval.

Stages \ Patient No.	1	2	3	4	5	6	7	8	9	10	11	12	13	14	Mean	SD	SE	CV	95% CI	
																			Lower	Upper
0-1	-1.32191841	-2.89002802	-1.47606503	-2.70746189	-5.61115718	-2.10617742	-3.02791565	-3.28528486	-1.57109188	-2.90273994	-2.38024107	-1.31972746	-2.39508042	-2.66182664	-2.546908277	1.102448	0.294642	-43.2857348	-3.15742361	-1.93639295
1-2	0.12756514	-0.10435539	-0.88619043	0.09266298	-0.05284303	0.06923522	0.14957508	-0.3126644	-1.11127962	0.37899108	0.70287916	0.19391175	-0.24318072	0.08142052	-0.065305191	0.469458	0.125468	-718.867417	-0.32528222	0.19467184
2-3	0.11720025	-0.33488876	0.0279316	0.16890323	0.18593188	0.20528351	0.14167265	0.26417127	0.15403746	0.14070236	0.12861905	0.1246802	0.25116061	0.14607164	0.122962641	0.144147	0.038525	117.2282295	0.04313673	0.20278855
3-6	0.01227728	-0.07463814	0.08581631	-0.01546649	-0.04175251	-0.10993108	-0.04783129	0.02843304	-0.14682724	-0.14673787	-0.13237202	-0.02741414	-0.10527766	-0.16811088	-0.063559477	0.075652	0.020219	-119.024861	-0.10545393	-0.02166503
6-9	-0.01492519	-0.03095893	-0.04800093	-0.01755032	-0.06819398	-0.04463854	-0.02103607	-0.03351833	-0.03467688	-0.06422188	-0.02050826	-0.04176701	-0.04387702	-0.02446492	-0.036309876	0.016549	0.004423	-45.5775439	-0.04547449	-0.02714526

Supplementary Table 7. Intracranial elastance (IE) on the brain tissue of patients from before shunting to 15 months after shunting. The unit of all values is cm H2O/ml. The ICP changes after the sixth month was negligible. Hence, the elastance values measure to the sixth month. Stages 0-1 means the intracranial elastance between before shunting and the first month after shunting.

SD: standard deviation; SE: standard error; CV: coefficient of variation; CI: confidence of interval.

Patient No. / Stages	1	2	3	4	5	6	7	8	9	10	11	12	13	14	Mean	SD	SE	CV	95% CI	
																			Lower	Upper
0-1	0.05722	0.06450	0.06723	0.10465	0.06742	0.07662	0.08073	0.07112	0.05014	0.07468	0.06353	0.08122	0.05908	0.06460	0.07020	0.01330	0.00356	18.94892	0.06283	0.07756
1-2	0.14494	-0.10949	0.01152	-0.22202	0.15317	-0.13011	0.08424	-0.34286	0.07899	-0.32444	0.37400	-0.19398	0.12177	-0.34225	-0.04975	0.22080	0.05901	-443.8009	-0.17203	0.07252
2-3	0.02979	-0.33286	0.22800	-0.01856	0.00956	0.02266	0.02483	0.01171	-0.01404	0.03231	0.02857	-0.01826	0.00507	-0.02878	-0.00143	0.11388	0.03043	-7973.177	-0.06449	0.06163
3-6	-0.02973	0.02455	-0.00984	0.01727	-0.02426	-0.02588	0.41000	-0.01773	0.06139	-0.04091	0.17059	0.03307	0.29474	0.10429	0.06911	0.13533	0.03617	195.81079	-0.00583	0.14405

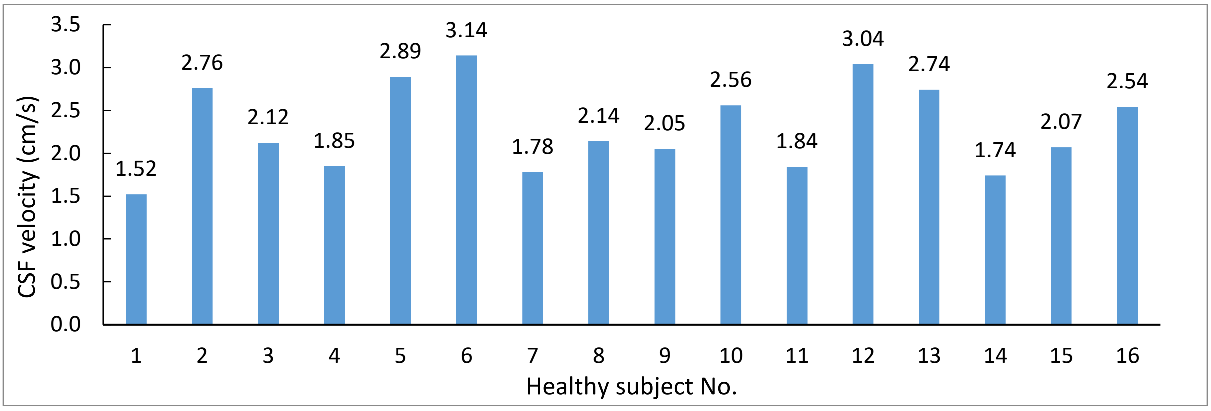

Supplementary Figure 1. CSF velocity in the cerebral aqueduct of healthy subjects. The mean, standard deviation, standard error, and coefficient of variation are 2.30 cm/s, 0.51, 0.13, and 22.21%, respectively. The upper and lower confidence interval are 2.55 and 2.04, respectively.

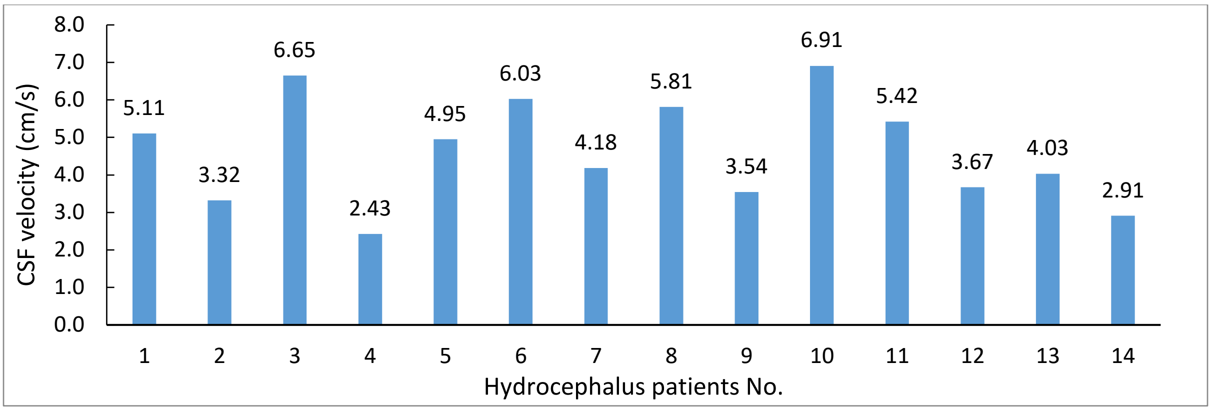

Supplementary Figure 2. CSF velocity in the cerebral aqueduct of hydrocephalus patients. The mean, standard deviation, standard error, and coefficient of variation are 4.64 cm/s, 1.41, 0.38, and 30.34%, respectively. The upper and lower confidence interval are 5.34 and 3.94, respectively.

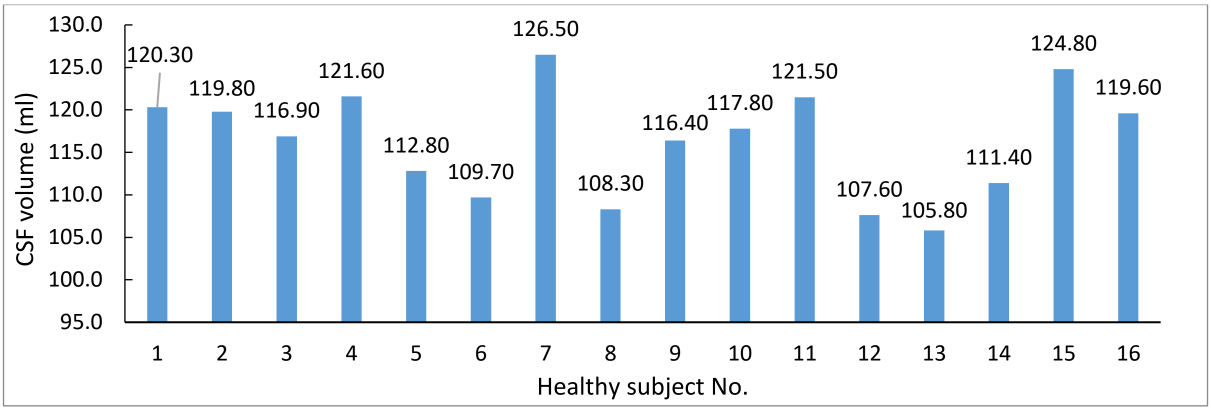

Supplementary Figure 3. CSF volume of healthy subjects. The mean, standard deviation, standard error, and coefficient of variation are 116.30 ml, 6.34, 1.59, and 5.45%, respectively. The upper and lower confidence interval are 119.45 and 113.15, respectively.

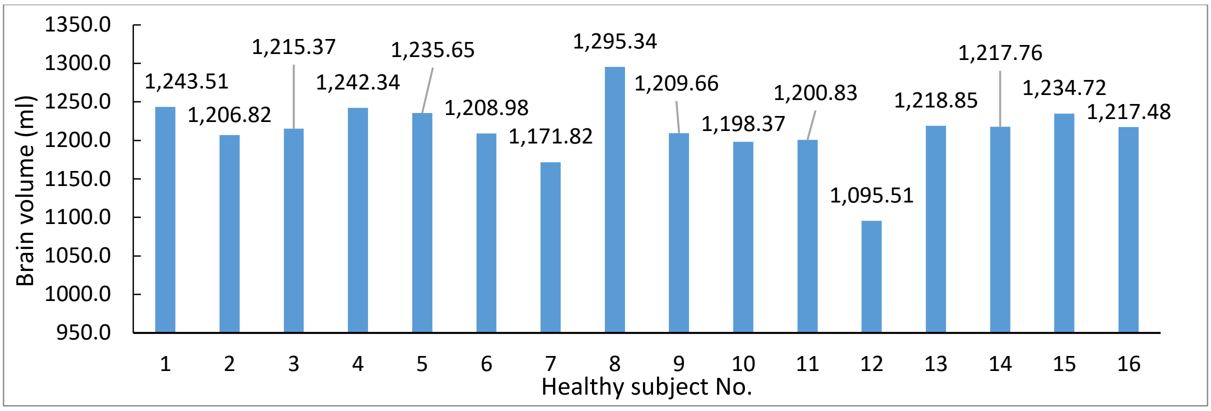

Supplementary Figure 4. The brain volume of healthy subjects. The mean, standard deviation, standard error, and coefficient of variation are 1,213.31 ml, 41.28, 10.32, and 3.40%, respectively. The upper and lower confidence interval are 1,233.84 and 1,192.78, respectively.

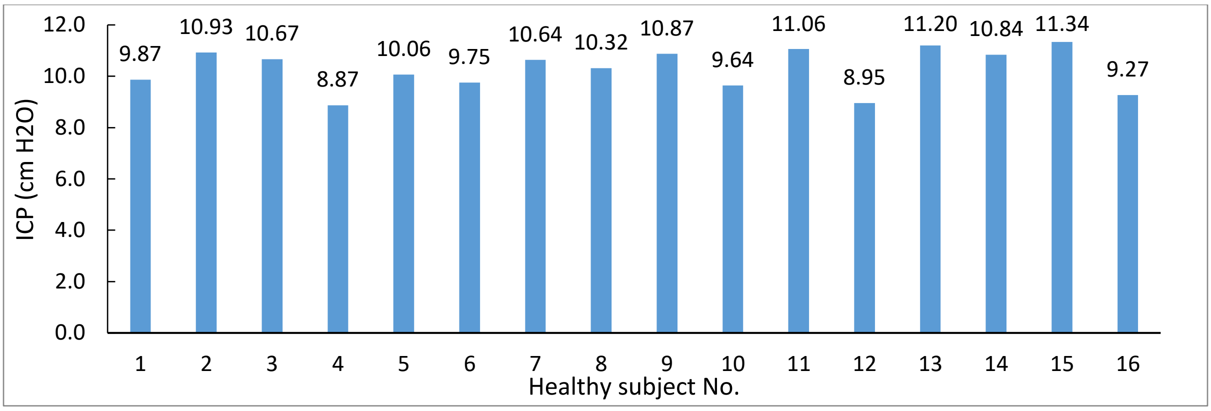

Supplementary Figure 5. ICP of healthy subjects. The mean, standard deviation, standard error, and coefficient of variation are 10.27 cm H_2O, 0.80, 0.20, and 7.80%, respectively. The upper and lower confidence interval are 10.67 and 9.87, respectively.

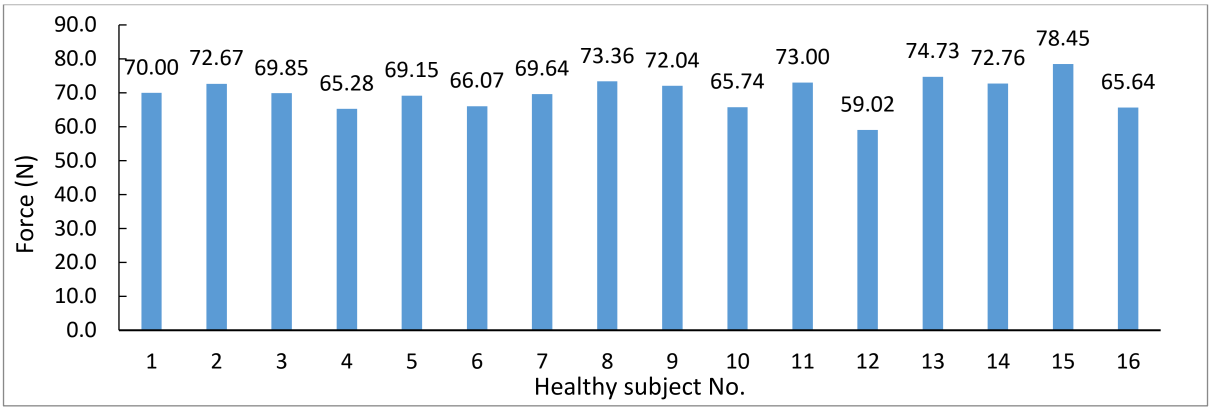

Supplementary Figure 6. Exerted force on the brain tissue of healthy subjects. The mean, standard deviation, standard error, and coefficient of variation are 69.84 N, 4.68, 1.17, and 6.70%, respectively. The upper and lower confidence interval are 72.17 and 67.51, respectively.
