# Supplementary material for: Long-term recovery behavior of brain tissue in hydrocephalus patients after shunting
Source: Commun Biol. 2022 Nov 8;5:1198. doi: 10.1038/s42003-022-04128-8 (PMC9640582; doi:10.1038/s42003-022-04128-8)
Supplement: Supplementary file 2 — Description of Additional Supplementary Files [file 42003_2022_4128_MOESM2_ESM.pdf]

Description of Additional Supplementary File

File name: Supplementary Data 1.

Description: CSF and brain volume and ICP changes of patients from before shunting to 15 months after shunting. CSF: cerebrospinal fluid; ICP: Intracranial pressure.
