# Supplementary material for: Long-term recovery behavior of brain tissue in hydrocephalus patients after shunting
Source: Commun Biol. 2022 Nov 8;5:1198. doi: 10.1038/s42003-022-04128-8 (PMC9640582; doi:10.1038/s42003-022-04128-8)
Supplement: Supplementary file 3 — Supplementary Data 1 [file 42003_2022_4128_MOESM3_ESM.docx]

| Time  No. of patient | Age (Male/Female) | Before shunting | | | | 1 Month after shunting | | | | 2 Months after shunting | | | | 3 Months after shunting | | | | 6 Months after shunting | | | | 9 Months after shunting | | | | 12 Months after shunting | | | | 15 Months after shunting | | | |
| --- | --- | --- | --- | --- | --- | --- | --- | --- | --- | --- | --- | --- | --- | --- | --- | --- | --- | --- | --- | --- | --- | --- | --- | --- | --- | --- | --- | --- | --- | --- | --- | --- | --- |
|  |  | CSF volume  (ml) | Brain volume  (ml) | ICP (cm H2O) | Force (N) | CSF volume  (ml) | Brain volume  (ml) | ICP (cm H2O) | Force (N) | CSF volume  (ml) | Brain volume  (ml) | ICP (cm H2O) | Force (N) | CSF volume  (ml) | Brain volume  (ml) | ICP (cm H2O) | Force (N) | CSF volume  (ml) | Brain volume  (ml) | ICP (cm H2O) | Force (N) | CSF volume  (ml) | Brain volume  (ml) | ICP (cm H2O) | Force (N) | CSF volume  (ml) | Brain volume  (ml) | ICP (cm H2O) | Force (N) | CSF volume  (ml) | Brain volume  (ml) | ICP (cm H2O) | Force (N) |
| 1 | 65 (Male) | 389.14 | 1104.61 | 25.12 | 179.02 | 136.80 | 1178.60 | 10.68 | 81.21 | 152.60 | 1199.40 | 12.97 | 83.86 | 138.50 | 1220.10 | 12.55 | 86.29 | 149.60 | 1242.60 | 12.22 | 86.56 | 141.50 | 1261.40 | 12.20 | 86.28 | 130.70 | 1275.50 | 12.07 | 85.32 | 130.20 | 1295.50 | 12.11 | 85.62 |
| 2 | 50 (Male) | 410.27 | 924.68 | 26.35 | 157.20 | 152.30 | 958.30 | 9.71 | 60.03 | 138.60 | 999.20 | 11.21 | 55.76 | 145.60 | 1027.30 | 8.88 | 46.35 | 123.60 | 1046.60 | 8.34 | 44.91 | 136.80 | 1067.10 | 8.32 | 44.28 | 122.40 | 1080.60 | 8.27 | 43.66 | 121.90 | 1100.50 | 8.19 | 42.55 |
| 3 | 68 (Female) | 425.67 | 1169.50 | 31.47 | 237.45 | 153.60 | 1257.90 | 13.18 | 106.96 | 137.10 | 1276.50 | 12.99 | 90.48 | 134.60 | 1289.80 | 12.42 | 90.85 | 146.80 | 1305.50 | 12.30 | 92.20 | 137.60 | 1319.40 | 12.28 | 91.53 | 130.50 | 1327.50 | 12.26 | 91.00 | 127.90 | 1347.80 | 12.32 | 91.53 |
| 4 | 54 (Male) | 372.80 | 1024.87 | 32.76 | 216.61 | 146.80 | 1081.40 | 9.11 | 63.56 | 135.90 | 1102.40 | 11.53 | 65.50 | 152.60 | 1121.00 | 11.22 | 68.65 | 130.60 | 1140.30 | 10.84 | 68.35 | 136.50 | 1160.30 | 10.82 | 68.00 | 128.60 | 1172.60 | 10.63 | 66.42 | 128.10 | 1196.30 | 10.62 | 66.37 |
| 5 | 72 (Male) | 408.91 | 1103.46 | 28.14 | 200.33 | 134.20 | 1126.70 | 9.62 | 69.93 | 146.80 | 1146.10 | 11.55 | 68.90 | 121.70 | 1165.80 | 11.31 | 72.57 | 138.60 | 1183.50 | 10.90 | 71.83 | 125.90 | 1198.40 | 10.84 | 70.81 | 119.90 | 1214.50 | 10.82 | 70.78 | 119.70 | 1230.40 | 10.68 | 69.18 |
| 6 | 59 (Female) | 414.27 | 984.51 | 29.67 | 188.45 | 161.20 | 1041.20 | 10.28 | 69.06 | 142.60 | 1060.40 | 12.70 | 70.38 | 129.80 | 1076.50 | 12.41 | 73.69 | 152.60 | 1092.60 | 11.82 | 71.92 | 136.50 | 1108.20 | 11.78 | 71.22 | 128.80 | 1124.50 | 11.75 | 71.24 | 128.60 | 1139.30 | 11.82 | 71.28 |
| 7 | 61 (Female) | 394.57 | 1154.91 | 32.61 | 242.98 | 122.32 | 1207.80 | 10.63 | 82.83 | 149.50 | 1228.10 | 12.92 | 85.87 | 134.60 | 1245.70 | 12.55 | 88.36 | 133.60 | 1263.10 | 12.14 | 87.53 | 139.80 | 1281.70 | 12.11 | 87.14 | 132.50 | 1299.50 | 12.06 | 87.11 | 132.10 | 1313.30 | 12.10 | 86.92 |
| 8 | 54 (Male) | 431.67 | 1013.24 | 31.27 | 204.41 | 146.80 | 1052.70 | 11.01 | 74.78 | 142.60 | 1069.80 | 12.45 | 69.43 | 131.50 | 1086.60 | 12.32 | 73.87 | 145.60 | 1099.80 | 12.07 | 74.24 | 137.80 | 1117.40 | 12.02 | 73.65 | 129.50 | 1139.70 | 12.00 | 74.23 | 129.10 | 1156.30 | 12.06 | 74.37 |
| 9 | 66 (Female) | 427.18 | 1162.37 | 27.64 | 207.28 | 143.60 | 1226.70 | 13.42 | 106.21 | 129.80 | 1247.80 | 12.33 | 82.76 | 152.60 | 1266.40 | 12.01 | 85.63 | 142.50 | 1284.40 | 11.39 | 82.98 | 136.50 | 1302.30 | 11.35 | 82.36 | 128.90 | 1324.60 | 11.24 | 82.05 | 128.40 | 1345.20 | 11.33 | 82.73 |
| 10 | 67 (Female) | 402.19 | 1121.67 | 29.14 | 210.87 | 154.60 | 1166.70 | 10.65 | 80.16 | 145.60 | 1185.50 | 13.57 | 87.29 | 132.60 | 1199.60 | 13.15 | 89.27 | 145.80 | 1213.10 | 12.61 | 87.29 | 138.60 | 1227.90 | 12.54 | 86.34 | 131.40 | 1251.80 | 12.35 | 85.74 | 130.90 | 1267.40 | 12.34 | 85.30 |
| 11 | 55 (Female) | 449.21 | 1095.20 | 30.16 | 213.10 | 142.60 | 1151.40 | 10.68 | 79.34 | 152.60 | 1169.90 | 14.42 | 92.34 | 138.60 | 1195.60 | 14.02 | 95.64 | 135.20 | 1211.00 | 13.44 | 93.61 | 140.20 | 1227.40 | 13.42 | 93.27 | 134.20 | 1242.80 | 13.29 | 92.56 | 133.80 | 1257.50 | 13.34 | 92.63 |
| 12 | 70 (Female) | 421.61 | 1023.46 | 33.18 | 219.09 | 139.80 | 1132.50 | 10.29 | 75.18 | 126.50 | 1153.70 | 12.87 | 79.29 | 149.50 | 1173.40 | 12.45 | 81.75 | 136.80 | 1193.20 | 12.03 | 81.21 | 128.60 | 1212.50 | 11.94 | 80.40 | 120.80 | 1230.70 | 11.94 | 80.80 | 120.60 | 1242.20 | 11.82 | 79.13 |
| 13 | 59 (Male) | 386.17 | 1062.74 | 25.84 | 177.17 | 143.80 | 1102.50 | 11.52 | 81.94 | 156.20 | 1120.10 | 13.03 | 77.66 | 134.50 | 1139.20 | 12.92 | 82.46 | 132.60 | 1155.50 | 12.36 | 80.74 | 141.50 | 1171.50 | 12.31 | 80.04 | 130.70 | 1190.70 | 12.16 | 79.41 | 130.10 | 1208.20 | 12.14 | 79.03 |
| 14 | 62 (Male) | 446.18 | 1006.54 | 29.81 | 193.58 | 139.70 | 1053.70 | 10.01 | 68.05 | 132.60 | 1070.30 | 12.44 | 69.40 | 146.50 | 1088.40 | 12.04 | 72.04 | 139.50 | 1105.10 | 11.31 | 69.24 | 131.60 | 1124.50 | 11.27 | 68.76 | 130.20 | 1139.40 | 11.22 | 68.48 | 129.70 | 1154.70 | 11.32 | 68.73 |
| Mean | 61.6 | 412.85 | 1067.98 | 29.51 | 203.40 | 144.15 | 1124.15 | 10.77 | 78.52 | 142.07 | 1144.94 | 12.64 | 77.07 | 138.80 | 1163.96 | 12.16 | 79.10 | 139.53 | 1181.16 | 11.70 | 78.04 | 136.39 | 1198.57 | 11.66 | 77.44 | 128.51 | 1215.31 | 11.58 | 77.06 | 127.94 | 1232.47 | 11.59 | 76.81 |
