# Supplementary material for: Long-term recovery behavior of brain tissue in hydrocephalus patients after shunting
Source: Commun Biol. 2022 Nov 8;5:1198. doi: 10.1038/s42003-022-04128-8 (PMC9640582; doi:10.1038/s42003-022-04128-8)
Supplement: Supplementary file 4 — Reporting Summary [file 42003_2022_4128_MOESM4_ESM.pdf]

nature research

Corresponding author(s): Bakhtiar Yamini

Last updated by author(s): Jul 13, 2022

Reporting Summary

Nature Research wishes to improve the reproducibility of the work that we publish. This form provides structure for consistency and transparency in reporting. For further information on Nature Research policies, see our Editorial Policies and the Editorial Policy Checklist.

Statistics

For all statistical analyses, confirm that the following items are present in the figure legend, table legend, main text, or Methods section.

n/a	Confirmed	
☐	☒	The exact sample size (n) for each experimental group/condition, given as a discrete number and unit of measurement
☐	☒	A statement on whether measurements were taken from distinct samples or whether the same sample was measured repeatedly
☐	☒	The statistical test(s) used AND whether they are one- or two-sided<br>*Only common tests should be described solely by name; describe more complex techniques in the Methods section.*
☐	☒	A description of all covariates tested
☐	☒	A description of any assumptions or corrections, such as tests of normality and adjustment for multiple comparisons
☐	☒	A full description of the statistical parameters including central tendency (e.g. means) or other basic estimates (e.g. regression coefficient) AND variation (e.g. standard deviation) or associated estimates of uncertainty (e.g. confidence intervals)
☐	☒	For null hypothesis testing, the test statistic (e.g. F, t, r) with confidence intervals, effect sizes, degrees of freedom and P value noted<br>*Give P values as exact values whenever suitable.*
☒	☐	For Bayesian analysis, information on the choice of priors and Markov chain Monte Carlo settings
☐	☒	For hierarchical and complex designs, identification of the appropriate level for tests and full reporting of outcomes
☐	☒	Estimates of effect sizes (e.g. Cohen's d, Pearson's r), indicating how they were calculated

Our web collection on statistics for biologists contains articles on many of the points above.

Software and code

Policy information about availability of computer code

Data collection	We have used the MRI files of patients, and we prepared all of these data according to the ethical committee of Tarish Hospital, and the 1964 Helsinki declaration and its later amendments
Data analysis	We have used ADINA 8.3 (Adina R&D Inc., Watertown MA, USA) software for data analysis. However, we used the routine toolbar of this software and we didn't use any specific codes. Moreover, we used SPSS for statistical analysis.

For manuscripts utilizing custom algorithms or software that are central to the research but not yet described in published literature, software must be made available to editors and reviewers. We strongly encourage code deposition in a community repository (e.g. GitHub). See the Nature Research guidelines for submitting code & software for further information.

Data

Policy information about availability of data

All manuscripts must include a data availability statement. This statement should provide the following information, where applicable:

- Accession codes, unique identifiers, or web links for publicly available datasets
- A list of figures that have associated raw data
- A description of any restrictions on data availability

All data used in this manuscript are publicly available and can be found at original publications (main text or supplementary materials) or repositories. The MRI files of subjects, however, contain some identifying information of patients and normal subjects, and cannot be made publicly available. The data are available from the corresponding author.

Field-specific reporting

Please select the one below that is the best fit for your research. If you are not sure, read the appropriate sections before making your selection.

☒ Life sciences ☐ Behavioural & social sciences ☐ Ecological, evolutionary & environmental sciences

For a reference copy of the document with all sections, see nature.com/documents/nr-reporting-summary-flat.pdf

Life sciences study design

All studies must disclose on these points even when the disclosure is negative.

Sample size	14 adult communicating hydrocephalus patients and 16 healthy subject
Data exclusions	14 of 38 adult communicating hydrocephalus patients did not have shunt failure and the neurosurgeons did not also need to change the adjustment of the shunt valve during the 15 months. With regard to the goal of the present study, the data of these 14 patients with successful shunting without any changes in valve adjustment were used in the present study. These patients did not have any history of other central nervous system disorders and/or surgeries. It should be noted that we didnot work on patients with failed shunting.
Replication	All attempt at replication were successful statistically
Randomization	Allocation of subjects was random
Blinding	Prior to scanning, written informed consent was obtained from all the volunteers. All MRI data were anonymized prior to transfer to operators for analysis.

Reporting for specific materials, systems and methods

We require information from authors about some types of materials, experimental systems and methods used in many studies. Here, indicate whether each material, system or method listed is relevant to your study. If you are not sure if a list item applies to your research, read the appropriate section before selecting a response.

Materials & experimental systems

n/a	Involved in the study	
☒	☐	Antibodies
☒	☐	Eukaryotic cell lines
☒	☐	Palaeontology and archaeology
☒	☐	Animals and other organisms
☐	☒	Human research participants
☒	☐	Clinical data
☒	☐	Dual use research of concern

Methods

n/a	Involved in the study	
☐	☐	ChIP-seq
☐	☐	Flow cytometry
☐	☒	MRI-based neuroimaging

Human research participants

Policy information about studies involving human research participants

Population characteristics	Among 38 adult communicating hydrocephalus patients, 14 patients did not need shunt revisions during 15 months. We studied the MRI images of 16 healthy subjects and 14 communicating hydrocephalus patients in pre-shunting and 7 stages after shunting. The present study is centered on calculation of the changes in volume, strain, and creep of CSF and brain, as well as ICP, exerted force on the brain tissue, brain stiffness, and intracranial elastance (IE) non-invasively using fluid-structure interaction (FSI) simulation to evaluate the brain recovery behavior of hydrocephalus patients over 15 months after shunting. It should be noted that there was no history of other neurological problems in these patients. Medtronic ventriculoperitoneal shunt was used to treat all the patients. Tarish Neurosurgical team diagnose and treated these patients.
Recruitment	Body mass index and age of patients were 24.1-28.7 kg/m2 and 50-72 years, respectively. The corresponding values for healthy subjects were 22.6-27.3 kg/m2 and 44-67 years, respectively.
Ethics oversight	The data access and ethics committees of Tarish Hospital, and the 1964 Helsinki declaration and its later amendments.

Note that full information on the approval of the study protocol must also be provided in the manuscript.

ChIP-seq

Data deposition

☐ Confirm that both raw and final processed data have been deposited in a public database such as GEO.

☒ Confirm that you have deposited or provided access to graph files (e.g. BED files) for the called peaks.

Data access links<br>*May remain private before publication.*	We create our data base with regards to all policy of our ethical approval and data access committee and we share it in the manuscript except for MRI images. According to this policy for accessing the data should contact with corresponding author for the MRI images.
Files in database submission	We create our data base with regards to all policy of our ethical approval and data access committee and we share it in the manuscript except for MRI images. According to this policy for accessing the data should contact with corresponding author for the MRI images.
Genome browser session<br>(e.g. UCSC)	We did not have any genome in our study.

Methodology

Replicates	First, the CSF velocity diagram for each patient and healthy subjects in each step was calculated using FSI simulation and was compared with the CSF velocity in aqueduct of Sylvius which was measured experimentally using the CINE phase-contrast magnetic resonance imaging (CINE PC-MRI). All experimental test were repeated five times. In addition the calculated ICP also compared with experimental ICP values. The statistical analysis confirmed the reliability and significantly of the data base. Yo can see all details in the workflow of Fig. 6.
Sequencing depth	All experimental testing was repeated five times. It should be noted the time and length of tests were not important and effective in the tests.
Antibodies	No antibodies were used in the tests.
Peak calling parameters	We reported the peak of each parameter in a cardiac cycle. Then we reported the mean, SD, SE, CV, and CI of the peak value of that parameter for all subjects.
Data quality	For insurance about correctness of our data, we have compared our computer simulation data with the experimental data (CINE PC-MRI and ICP monitoring).
Software	DICOM files obtained from MRI of each patient were transferred to Mimics software v13.1 to prepare the points cloud that the points in the cloud were voxel centres. The point clouds of the head substructures (SAS, brain tissue, and ventricular system) were produced for each patient and transferred to CATIA v5.R21 for 3D geometrical modeling. After creating 3D geometrical models of the head of patients separately (Fig. 1), the models were transferred to ADINA 8.3 (Adina R&D Inc., Watertown MA, USA) for meshing (Fig. 1) and analysis. It should be noted that we only used the routine toolbars of these softwares and we did not write any programming. Moreover, we use SPSS software to statistical analysis.

Flow Cytometry

Plots

Confirm that:

☐ The axis labels state the marker and fluorochrome used (e.g. CD4-FITC).

☒ The axis scales are clearly visible. Include numbers along axes only for bottom left plot of group (a 'group' is an analysis of identical markers).

☒ All plots are contour plots with outliers or pseudocolor plots.

☒ A numerical value for number of cells or percentage (with statistics) is provided.

Methodology

Sample preparation	30 patients were recruited: 14 patients and 16 healthy subjects.
Instrument	MRI machine and a workstation for simulations
Software	DICOM files obtained from MRI of each patient were transferred to Mimics software v13.1 to prepare the points cloud that the points in the cloud were voxel centres. The point clouds of the head substructures (SAS, brain tissue, and ventricular system) were produced for each patient and transferred to CATIA v5.R21 for 3D geometrical modeling. After creating 3D geometrical models of the head of the patients separately (Fig. 1), the models were transferred to ADINA 8.3 (Adina R&D Inc., Watertown MA, USA) for meshing (Fig. 1) and analysis. It should be noted that we only used the routine toolbars of these softwares and we did not write any programming. Moreover, we use SPSS software to statistical analysis.
Cell population abundance	We did not use Cell in our study

Gating strategy	We did not use Gating strategy in our study

☐ Tick this box to confirm that a figure exemplifying the gating strategy is provided in the Supplementary Information.

Magnetic resonance imaging

Experimental design

Design type	We used event-relevant
Design specifications	Each test was repeated for 5 times. Scanning was performed using a 3 Tesla MRI system (Magnetom Trio, Siemens, Erlangen, Germany), with the acquisition time of 4.5 minutes.
Behavioral performance measures	Each test was repeated for 5 times. We calculated the mean, SD, SE, confidence interval and coefficient of variation.

Acquisition

Imaging type(s)	We used structural imaging (static MRI) - cine-MRI
Field strength	A 3 Tesla (Magnetom Trio, Siemens, Erlangen, Germany) was used for generating MRI data.
Sequence & imaging parameters	MRI includes a cardiac-gated PC to quantify CSF velocity and an axial T2 weighted image (T2WI). The time to echo/repetition time, field of view, slice intervals/slice thickness, and flip angle for axial T2WI equaled 117/4,000 ms, 220×220 mm, 1.8/6 mm, and 90°, respectively. The corresponding values for PC-MRI equaled 7/21 ms, 160×160 mm, 1.2/6 mm, and 10°, respectively. The acquisition times for axial T2WI and PC-MRI were 150 sec and 270 sec, respectively. It should be noted that velocity encoding in PC-MRI was 15 cm/s. A 3 Tesla (Magnetom Trio, Siemens, Erlangen, Germany) was used for generating MRI data.
Area of acquisition	The pixel velocity in CSF areas was corrected by subtracting the average velocity of solid brain tissue in a nearly 220×220 mm fot T2 and 160×160 mm for cine PC.

Diffusion MRI ☐ Used ☒ Not used

Preprocessing

Preprocessing software	DICOM files obtained from MRI of each patient were transferred to Mimics software v13.1 to prepare the points cloud that the points in the cloud were voxel centres. The point clouds of the head substructures (SAS, brain tissue, and ventricular system) were produced for each patient and transferred to CATIA v5.R21 for 3D geometrical modeling. After creating 3D geometrical models of the head of the patients separately (Fig. 1), the models were transferred to ADINA 8.3 (Adina R&D Inc., Watertown MA, USA) for meshing (Fig. 1) and analysis (Fig. 6d, e) . It should be noted that we only used the routine toolbars of the software and we did not write any additional programming.
Normalization	The inlet and outlets boundary conditions were calculated with the superposition of a constant value of flow rate and the normalized pulsatile profile of the blood flow rate in the basilar artery, which was measured with the CINE PC-MRI for all hydrocephalus patients. The process of normalization were calculated using MATLAB software.
Normalization template	We only used the MATLAB toolbars for normalizing the flow rate function of the blood for inlet and outlets boundary conditions
Noise and artifact removal	We did not use the noise removing process.
Volume censoring	We did not use volume censoring

Statistical modeling & inference

Model type and settings	The results of Shapiro-Wilk tests confirmed the normal distributions in all datasets. The parametric ANOVA multiple comparisons were performed using IBM SPSS software to compare all data in the various conditions and different stages after shunting as well as between treated patients (15 months after shunting) and healthy subjects. The homogeneity evaluation of the variance test showed equal variances. To compare the datasets after ANOVA, Tukey's posthoc test was performed for pairwise comparison. We also used Student's t-test to compare the results of CINE PC-MRI and FSI simulation for CSF velocity in the cerebral aqueduct. T and F were the test statistics for Student's t-test and ANOVA, respectively. Pearson correlation coefficient was performed to assess the relationship of intracranial elastance with ICP. In addition, all values were stated as mean ± standard error, and P-value=0.05 was defined as the
Effect(s) tested	We did not need to assess the effects test

Specify type of analysis: ☒ Whole brain ☐ ROI-based ☐ Both

Statistic type for inference (See Eklund et al. 2016)	We have not needed to assess voxel/cluster-wise.
Correction	We have not needed to assess the correction.

Models & analysis

n/a	Involved in the study
[x]	[ ] Functional and/or effective connectivity
[ ]	[x] Graph analysis
[ ]	[x] Multivariate modeling or predictive analysis

Graph analysis	Pearson correlation coefficient was used to describe the relationship of elstance with ICP for all 14 patients.
Multivariate modeling and predictive analysis	*Specify independent variables, features extraction and dimension reduction, model, training and evaluation metrics.*
